# Multivariate Analysis and Visualization of Splicing Correlations in Single-Gene Transcriptomes

**DOI:** 10.1186/1471-2105-8-16

**Published:** 2007-01-18

**Authors:** Mark C Emerick, Giovanni Parmigiani, William S Agnew

**Affiliations:** 1 Department of Physiology, Johns Hopkins Medical School, Baltimore, MD 21205 USA; 2 Departments of Oncology, Zoology, Johns Hopkins Medical School, and Biostatistics, Johns Hopkins Bloomberg School of Public Health, Baltimore, MD 21205 USA; 3 Departments of Physiology and Neuroscience, Johns Hopkins Medical School, Baltimore, MD 21205 USA

## Abstract

**Background:**

RNA metabolism, through 'combinatorial splicing', can generate enormous structural diversity in the proteome. Alternative domains may interact, however, with unpredictable phenotypic consequences, necessitating integrated RNA-level regulation of molecular composition. Splicing correlations within transcripts of single genes provide valuable clues to functional relationships among molecular domains as well as genomic targets for higher-order splicing regulation.

**Results:**

We present tools to visualize complex splicing patterns in full-length cDNA libraries. Developmental changes in pair-wise correlations are presented vectorially in '*clock plots' *and linkage grids. Higher-order correlations are assessed statistically through Monte Carlo analysis of a log-linear model with an empirical-Bayes estimate of the true probabilities of observed and unobserved splice forms. Log-linear coefficients are visualized in a '*spliceprint,' *a signature of splice correlations in the transcriptome. We present two novel metrics: the *linkage change index*, which measures the directional change in pair-wise correlation with tissue differentiation, and the *accuracy index*, a very simple goodness-of-fit metric that is more sensitive than the integrated squared error when applied to sparsely populated tables, and unlike chi-square, does not diverge at low variance. Considerable attention is given to sparse contingency tables, which are inherent to single-gene libraries.

**Conclusion:**

Patterns of splicing correlations are revealed, which span a broad range of interaction order and change in development. The methods have a broad scope of applicability, beyond the single gene – including, for example, multiple gene interactions in the complete transcriptome.

## Background

Through alternative splicing at multiple sites, a single transcriptional unit may give rise to a complex array of isoforms – a 'mini transcriptome,' or single-gene transcriptome (SGT). Considerable effort is being invested to assemble a genome-wide compendium of sites of transcript and peptide variations [1-6], the guiding principle being that combinatorial splicing may profoundly expand the proteome, and consequently the phenotypic repertoire, without increasing the number of structural genes [7].

Such phenotypic elaboration may arise through simple combinations of individual 'modular' domains, or through cooperative effects from multiple variable domains that interact functionally [8]. The latter complication imposes significant regulatory demands – for the efficient selection of combinations of viable combinations – which may in part underly the expansion of the non-coding genome [9,10]. To understand gene function more fully, we must determine how all the possible alternative domains are actually combined by the RNA splicing process into working molecules. This requires structural analysis of large numbers of full-length transcripts expressed from *each *gene, approaching numbers currently available in whole-genome full-length cDNA libraries derived from whole-genomes [11-16] to ensure adequate representation of the less abundant variants.

EST, microarray, and proteomics methodologies for large-scale alternative splicing surveys share the limitation that by sampling fragments of macromolecules they cannot capture most intramolecular linkage information. That is, they primarily yield marginal splicing frequencies but not correlations. The latter are invaluable for discerning the tissue- and cell-specific functional differentiation of splice variants [4,17]. Full-length cDNAs, by contrast, provide diaries of intramolecular splicing choices. We have developed robust methods for production of full-length, nonrecombinant, statistically representative single-gene libraries (SGLs) [18]. An SGL is a 'vertical' sample of the transcriptome, representing its basic building block, the SGT.

Initial analyses of moderate-scale SGLs [8,18,19] reveal fascinating developmental changes in both the extent and pattern of splicing linkage. These represent developmental changes in the regulatory programs that establish the selection rules for combining variable domains into functional ensembles, thus establishing the molecular phenotype. Recent developments in production of large-scale full-length cDNA libraries and high-throughput sequencing techniques [20,21] promise to provide this information at high resolution and on a meaningful scale.

As large-scale SGT data become available, reliable statistical methods will be required to discern potentially complex splicing interactions. Two features of such data have direct relevance to the present work: (1) high-order splicing correlations, which reflect high-order functional interactions within single molecules, and (2) very sparse representation of the complete configuration space, which presents a fundamental challenge to statistical analysis. Data sparseness results from a They typical situation where several alternative splice loci have low-frequency variations, rendering a substantial fraction of splice combinations as simply improbable.

Here we present tools for statistical analysis of high order splicing correlations in full-length SGLs

A thorough statistical analysis of alternative splicing (1) begins with full-length cDNA libraries that are unbiased, representative samples of the mRNA populations, (2) presents a complete discussion of marginal splicing frequencies as well as correlations, including tissue-to-tissue variations in these parameters, and (3) employs a stochastic splicing model where the observed data is a likely outcome. We present tools for analysis of splicing correlations in full-length SGLs, with careful attention to handling of sparse contingency tables. We provide simple, novel graphical visualizations that accentuate coordinated changes within groups of variable loci. The data – human low voltage-activated calcium channel gene (CACNA1G) SGLs from fetal and adult whole brain – derive from recent work [8], where we discuss in detail marginal splicing frequencies and their developmental changes, as well as splicing correlations, effects on ion channel function of alternative splicing at individual alternative domains, and high-order, non-additive functional interactions among multiple alternative domains. The present work builds on that material, presenting a detailed exposition of the statistical model, which was beyond the scope of that publication, a thorough analysis of splicing correlations, and visual tools that will prove generally useful in studies of splicing linkages. Since our focus is correlations, we limit our discussion of marginal frequencies to the extent to which it informs our understanding of correlations. Though we employ a particular dataset and splicing model for the sake of illustration, these analytical tools are not model-specific and are generally applicable to other types of multivariate data.

## Methods

### CACNA1G Gene and SGL Data

**Figure 1:**
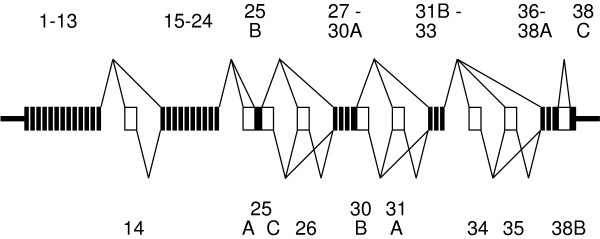
**CACNA1G Alternative Splicing Graph**. The CACNA1G gene comprises 38 known exons and 37 introns. All known exons or exon segments are shown. Lengths are not to scale. Introns are omitted. Black boxes depict constitutive (invariate) exons or exon segments, identified by the upper set of labels. Thin black segments on the ends are noncoding termini, corresponding to the alternative splice loci. White blocks depict segments corresponding to the alternative splice loci, labeled below the figure. Line segments joining the boxes, above and below, indicate potential alternative splices.

Table S1 presents the data for the present statistical analyses, listing the frequencies of full-length splice variants in whole-brain SGLs from two developmental stages of expression of the human calcium channel gene CACNA1G, described in reference 8. Marginal frequencies at the separate alternative loci calculated from this data are given in table S3, and are plotted in Figure S5 with confidence intervals. Figure 1 is a schematized CACNA1G gene structure illustrating the nine alternatively spliced exon segments (white blocks) and a splicing graph depicting the ways in exons may be joined by alternative splicing. There are 512 distinct structures, most of which have not been observed, and in fact may not be expressed.

### Definitions

An alternative splice *locus *is a categorical variable representing a region of the primary transcript that is subject to alternative splicing (we may omit the qualifier 'alternative' when the meaning is clear). We consider alternative splicing a stochastic process, so that every alternative sequence *configuration C*_*S*_, of any set of loci *S*, is a random variable with *splicing probability p*_*S*_(*C*_*S*_). We say that a locus *j *has a *strong splicing bias *if any *p*_*j*_(*C*_*j*_) approaches 1. For a single locus *j*, the configuration *C*_*j *_may be represented by an integer between 0 and *g*_*j *_- 1, where *g*_*j *_is the number of sequence alternatives, or *multiplicity*, of *j*. For a cassette exon, spliced in or out as a unit, it is often convenient to assign 1 to the insertion and 0 to the deletion, although the reverse may occasionally be more convenient – if the insertion is rare, for example. What defines a locus may also be flexible, depending on the purpose. An isolated cassette exon unambiguously defines a single binary locus, with multiplicity 2. Two adjacent alternatively spliced cassette exons, however, may be considered two loci with *g *= 2 or a single locus with *g *= 4. If the two exons are mutually exclusive then the best representation may be as a single locus with g = 3. Figure 1 illustrates the nine alternative loci in the CANCA1G gene.

If splicing at separate loci is independent [17,19,22], the expected frequency of each splice variant *v *is equal to its independent stochastic expectation

φv=∏j=1kfj(v),     (1)
 MathType@MTEF@5@5@+=feaafiart1ev1aaatCvAUfKttLearuWrP9MDH5MBPbIqV92AaeXatLxBI9gBaebbnrfifHhDYfgasaacH8akY=wiFfYdH8Gipec8Eeeu0xXdbba9frFj0=OqFfea0dXdd9vqai=hGuQ8kuc9pgc9s8qqaq=dirpe0xb9q8qiLsFr0=vr0=vr0dc8meaabaqaciaacaGaaeqabaqabeGadaaakeaaiiGacqWFgpGzdaWgaaWcbaGaemODayhabeaakiabg2da9maarahabaGaemOzay2aaSbaaSqaaiabdQgaQbqabaGccqGGOaakcqWG2bGDcqGGPaqkcqGGSaalcaWLjaGaaCzcamaabmaabaGaeGymaedacaGLOaGaayzkaaaaleaacqWGQbGAcqGH9aqpcqaIXaqmaeaacqWGRbWAa0Gaey4dIunaaaa@42AC@

where *f*_*j*_(*v*) is the marginal frequency of *C*_*j*_(*v*), the configuration of locus *j *in splice variant *v*. The number of possible full-length transcript variants is

NT=∏j=1kgj.     (2)
 MathType@MTEF@5@5@+=feaafiart1ev1aaatCvAUfKttLearuWrP9MDH5MBPbIqV92AaeXatLxBI9gBaebbnrfifHhDYfgasaacH8akY=wiFfYdH8Gipec8Eeeu0xXdbba9frFj0=OqFfea0dXdd9vqai=hGuQ8kuc9pgc9s8qqaq=dirpe0xb9q8qiLsFr0=vr0=vr0dc8meaabaqaciaacaGaaeqabaqabeGadaaakeaacqWGobGtdaWgaaWcbaGaemivaqfabeaakiabg2da9maarahabaGaem4zaC2aaSbaaSqaaiabdQgaQbqabaaabaGaemOAaOMaeyypa0JaeGymaedabaGaem4AaSganiabg+GivdGccqGGUaGlcaWLjaGaaCzcamaabmaabaGaeGOmaidacaGLOaGaayzkaaaaaa@3EA3@

Computations and illustrations were made in the *R *programming language [23].

### Mutual information methods

We quantify splicing linkage between a pair of loci *i *and *j *with the mutual information *I*(*i*, *j*), which measures the reduction in uncertainty about the configuration of one locus when that of the other is specified [24]: *I*(*i*, *j*) = *H*(*i*) + *H*(*j*) - *H*(*i*, *j*), where *H*(*i*) + *H*(*j*) is the expected entropy of *C*_*ij *_given independent splicing at the observed marginal frequencies, and *H*(*i*, *j*) = -∑_*ij*_*p*(*C*_*ij*_)·log *p*(*C*_*ij*_) is the observed entropy.

While mutual information is non-negative, it is useful to define a directed, or 'configuration-specific' mutual information, which may be negative. The sign gives the direction of correlation between a specific pair of configurations, called the *reference configurations*. For example, if we define the reference configuration at a pair of binary loci as the insertion at both loci, then a negative value means that insertion at one locus correlates with deletion at the other. The choice of reference configuration is arbitrary, and reversing the reference configuration for one locus simply reverses the sign of the configuration-specific mutual information.

The *dependency*, *D*(*i*|*j*) = *I*(*i*, *j*)/*H*(*i*), is the mutual information normalized to its maximum possible value, the total entropy of the 'dependent' locus, *i*. It measures the degree to which the independent variable is a predictor of the dependent variable: the one with lower marginal entropy has a higher dependency on the other.

### The Linkage change index

**Figure 2:**
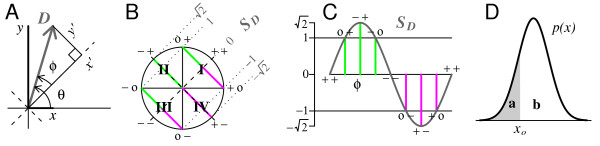
**Two metric indices introduced in this work**. A, The Linkage Vector, *D*, compares splicing correlation between two loci in two populations; B, The Linkage Change Index *S*_*D *_measures displacement of *D *from the zero-change axis (*x*'). C, *S*_*D *_is a sinusoidal function of the phase *φ *between *D *and *x*', the axis of zero linkage change. Positive values indicate an increasing positive or decreasing negative linkage change, and magnitude |*S*_*D*_| > 1 indicates a sign reversal (*c.f*. panel B). D, The Accuracy Index, A
 MathType@MTEF@5@5@+=feaafiart1ev1aaatCvAUfKttLearuWrP9MDH5MBPbIqV92AaeXatLxBI9gBamrtHrhAL1wy0L2yHvtyaeHbnfgDOvwBHrxAJfwnaebbnrfifHhDYfgasaacH8akY=wiFfYdH8Gipec8Eeeu0xXdbba9frFj0=OqFfea0dXdd9vqai=hGuQ8kuc9pgc9s8qqaq=dirpe0xb9q8qiLsFr0=vr0=vr0dc8meaabaqaciaacaGaaeqabaWaaeGaeaaakeaaimaacqWFaeFqaaa@3821@ = **a/b**, measures displacement of a point estimator *x*_0 _from the center of mass of a distribution *p*(*x*).

To quantify developmental changes in linkage we introduce the linkage change index, *S*_*D *_(Figure 2A–C). For a given pair of loci, we define the linkage vector *D *= (*x*, *y*), with components *x *– the splicing linkage in the fetal population, and *y *– the adult linkage. The difference *y *- *x *is a simple measure of the developmental change in linkage: it is zero when linkage is the same (*x *= *y*) at both stages and maximal (positive or negative) for a complete reversal of linkage (*x *= -*y*). Scaling to the length of *D *gives

SD=(y−x)/|D|=sin θ−cos θ=2sin φ
 MathType@MTEF@5@5@+=feaafiart1ev1aaatCvAUfKttLearuWrP9MDH5MBPbIqV92AaeXatLxBI9gBaebbnrfifHhDYfgasaacH8akY=wiFfYdH8Gipec8Eeeu0xXdbba9frFj0=OqFfea0dXdd9vqai=hGuQ8kuc9pgc9s8qqaq=dirpe0xb9q8qiLsFr0=vr0=vr0dc8meaabaqaciaacaGaaeqabaqabeGadaaakqaaeeqaaiabdofatnaaBaaaleaacqWGebaraeqaaOGaeyypa0JaeiikaGIaemyEaKNaeyOeI0IaemiEaGNaeiykaKIaei4la8IaeiiFaWNaemiraqKaeiiFaWhabaGaeyypa0dcbiGae83CamNae8xAaKMae8NBa4MaeeiiaaccciGae4hUdeNaeyOeI0Iae83yamMae83Ba8Mae83CamNaeeiiaaIae4hUdehabaGaeyypa0ZaaOaaaeaacqaIYaGmaSqabaGccqWFZbWCcqWFPbqAcqWFUbGBcqqGGaaicqGFgpGzaaaa@52AB@

where *θ *is the angle between *D *and the *x *axis (Figure 2A) and *φ *= *θ *- *π*/4 is the angle between *D *and the transformed *x *axis, *x*', representing unaltered linkage. In polar coordinates, *D *ranges in magnitude between about 1 and 1.4 times the larger of *x *and *y*, and has phase *φ*, the relative developmental change in linkage. Note that *sin φ *= *y*'/|*D*|, so *S*_*D *_is proportional to *y*', the perpendicular displacement of *D *from the *x*' axis.

*S*_*D *_is a more straightforward index of linkage change than either *φ *or *sin φ *(*c.f*. Figure 2B): *S*_*D *_is positive when splicing correlation becomes more positive or less negative with development (0 <*φ *<*π*, magenta lines in Figure 2B and 2C), and if the correlation actually reverses direction from negative to positive, then *S*_*D *_exceeds 1 (*π*/4 <*φ *< 3 *π*/4, quadrant II). Likewise, *S*_*D *_< 0 reflects increasing negative (red lines), or decreasing positive correlation, and *S*_*D *_< -1 means the linkage reverses, from positive to negative (quadrant IV). Figure 2C plots *S*_*D *_as a function of the phase *φ*, annotated corresponding to Figure 2B.

### Assessing higher-order linkages with log-linear models

Given a table of frequencies for *N*_*T *_splice variants (Table S1), it is natural to arrange the data in a *k*-dimensional contingency table with *k *variables (*j *= 1,..., *k*) of *g*_*j *_categories (*C*_*j *_= 0,..., *g*_*j *_- 1) each. This simplifies calculation of marginal frequencies. We fit the complete contingency table to a log-linear model [25], giving the log-frequency of each splice variant as a sum of coefficients *u*_*S*_(*C*_*S*_), which measure the extent of mutual correlation among a set of loci *S *with configuration *C*_*S*_. The most complete, or *saturated*, log-linear model is:

log p(C123...k)=grand meanindependencemain effects⋯order k-1u+u1(C1)+u2(C2)+⋯+uk(Ck)+u12(C12)+⋯+uk−1,k(Ck−1,k)+⋯+u123...k(C123...k)     (3)
 MathType@MTEF@5@5@+=feaafiart1ev1aaatCvAUfKttLearuWrP9MDH5MBPbIqV92AaeXatLxBI9gBaebbnrfifHhDYfgasaacH8akY=wiFfYdH8Gipec8Eeeu0xXdbba9frFj0=OqFfea0dXdd9vqai=hGuQ8kuc9pgc9s8qqaq=dirpe0xb9q8qiLsFr0=vr0=vr0dc8meaabaqaciaacaGaaeqabaqabeGadaaakeaafaqaaeqacaaabaqbaeGabyGaaaaabaacbiGae8hBaWMae83Ba8Mae83zaCMaeeiiaaIaemiCaaNaeiikaGIaem4qam0aaSbaaSqaaiabigdaXiabikdaYiabiodaZiabc6caUiabc6caUiabc6caUiabdUgaRbqabaGccqGGPaqkaeaacqGH9aqpaeaacqqGNbWzcqqGYbGCcqqGHbqycqqGUbGBcqqGKbazcqqGGaaicqqGTbqBcqqGLbqzcqqGHbqycqqGUbGBaeaaaeaacqqGPbqAcqqGUbGBcqqGKbazcqqGLbqzcqqGWbaCcqqGLbqzcqqGUbGBcqqGKbazcqqGLbqzcqqGUbGBcqqGJbWycqqGLbqzaeaaaeaacqqGTbqBcqqGHbqycqqGPbqAcqqGUbGBcqqGGaaicqqGLbqzcqqGMbGzcqqGMbGzcqqGLbqzcqqGJbWycqqG0baDcqqGZbWCaeaaaeaacqWIVlctaeaaaeaacqqGVbWBcqqGYbGCcqqGKbazcqqGLbqzcqqGYbGCcqqGGaaicqqGRbWAcqqGTaqlcqaIXaqmaeaaaaaabaqbaeaabyGaaaaabaaabaaabaaabaGaemyDauhabaGaey4kaScabaGaemyDau3aaSbaaSqaaiabigdaXaqabaGccqGGOaakcqWGdbWqdaWgaaWcbaGaeGymaedabeaakiabcMcaPiabgUcaRiabdwha1naaBaaaleaacqaIYaGmaeqaaOGaeiikaGIaem4qam0aaSbaaSqaaiabikdaYaqabaGccqGGPaqkcqGHRaWkcqWIVlctcqGHRaWkcqWG1bqDdaWgaaWcbaGaem4AaSgabeaakiabcIcaOiabdoeadnaaBaaaleaacqWGRbWAaeqaaOGaeiykaKcabaGaey4kaScabaGaemyDau3aaSbaaSqaaiabigdaXiabikdaYaqabaGccqGGOaakcqWGdbWqdaWgaaWcbaGaeGymaeJaeGOmaidabeaakiabcMcaPiabgUcaRiabl+UimjabgUcaRiabdwha1naaBaaaleaacqWGRbWAcqGHsislcqaIXaqmcqGGSaalcqWGRbWAaeqaaOGaeiikaGIaem4qam0aaSbaaSqaaiabdUgaRjabgkHiTiabigdaXiabcYcaSiabdUgaRbqabaGccqGGPaqkaeaacqGHRaWkaeaacqWIVlctaeaacqGHRaWkaeaacqWG1bqDdaWgaaWcbaGaeGymaeJaeGOmaiJaeG4mamJaeiOla4IaeiOla4IaeiOla4Iaem4AaSgabeaakiabcIcaOiabdoeadnaaBaaaleaacqaIXaqmcqaIYaGmcqaIZaWmcqGGUaGlcqGGUaGlcqGGUaGlcqWGRbWAaeqaaOGaeiykaKcaaaaacaWLjaGaaCzcamaabmaabaGaeG4mamdacaGLOaGaayzkaaaaaa@C91E@

where subscripts refer to alternative splice loci. Each full-length splice variant C_123...*k *_has a unique equation (3), giving *N*_*T *_equations in all. The saturated model has a term for every possible subset of loci plus an intercept, *u*, with ∑j=0k(kj)
 MathType@MTEF@5@5@+=feaafiart1ev1aaatCvAUfKttLearuWrP9MDH5MBPbIqV92AaeXatLxBI9gBaebbnrfifHhDYfgasaacH8akY=wiFfYdH8Gipec8Eeeu0xXdbba9frFj0=OqFfea0dXdd9vqai=hGuQ8kuc9pgc9s8qqaq=dirpe0xb9q8qiLsFr0=vr0=vr0dc8meaabaqaciaacaGaaeqabaqabeGadaaakeaadaaeWaqaamaabmaabaqbaeqabiqaaaqaaiabdUgaRbqaaiabdQgaQbaaaiaawIcacaGLPaaaaSqaaiabdQgaQjabg2da9iabicdaWaqaaiabdUgaRbqdcqGHris5aaaa@37B0@ terms in all. With all binary loci, the saturated model has 2^*k *^equations of 2^*k *^terms each. An unsaturated model is *hierarchical *if the presence of a *u*-term for any group of loci *S *implies a *u*-term for every subset of *S*.

For any locus *j *in a set of loci *S*, the sum of *u*_*S*_(*C*_*S*_) over all configurations of *j *is constrained to zero. Thus, for any *C*_*S *_and C′S
 MathType@MTEF@5@5@+=feaafiart1ev1aaatCvAUfKttLearuWrP9MDH5MBPbIqV92AaeXatLxBI9gBaebbnrfifHhDYfgasaacH8akY=wiFfYdH8Gipec8Eeeu0xXdbba9frFj0=OqFfea0dXdd9vqai=hGuQ8kuc9pgc9s8qqaq=dirpe0xb9q8qiLsFr0=vr0=vr0dc8meaabaqaciaacaGaaeqabaqabeGadaaakeaacuWGdbWqgaqbamaaBaaaleaacqWGtbWuaeqaaaaa@2F22@ differing only in the configuration of a binary locus *j*, *u*_*S*_(*C*_*S*_) = -*u*_*S*_(C′S
 MathType@MTEF@5@5@+=feaafiart1ev1aaatCvAUfKttLearuWrP9MDH5MBPbIqV92AaeXatLxBI9gBaebbnrfifHhDYfgasaacH8akY=wiFfYdH8Gipec8Eeeu0xXdbba9frFj0=OqFfea0dXdd9vqai=hGuQ8kuc9pgc9s8qqaq=dirpe0xb9q8qiLsFr0=vr0=vr0dc8meaabaqaciaacaGaaeqabaqabeGadaaakeaacuWGdbWqgaqbamaaBaaaleaacqWGtbWuaeqaaaaa@2F22@). If all loci in *S *are binary, then all terms *u*_*S*_(*C*_*S*_) have the same magnitude, |*u*_*S*_|. Working with all binary loci thus simplifies the analysis, but does not otherwise alter the capabilities of the method. We use normalized frequencies, rather than total counts, to allow direct comparison of populations of different sizes without rescaling

### Accuracy index

The 'accuracy,' A
 MathType@MTEF@5@5@+=feaafiart1ev1aaatCvAUfKttLearuWrP9MDH5MBPbIqV92AaeXatLxBI9gBamrtHrhAL1wy0L2yHvtyaeHbnfgDOvwBHrxAJfwnaebbnrfifHhDYfgasaacH8akY=wiFfYdH8Gipec8Eeeu0xXdbba9frFj0=OqFfea0dXdd9vqai=hGuQ8kuc9pgc9s8qqaq=dirpe0xb9q8qiLsFr0=vr0=vr0dc8meaabaqaciaacaGaaeqabaWaaeGaeaaakeaaimaacqWFaeFqaaa@3821@, measures the extent to which a point, *x*_0_, is centered within a distribution, *p*(*x*). This has the advantage of extreme simplicity: it is the ratio of areas **a **and **b **in Figure 2D, where **a **is always the smaller of the two areas ∑{*p*(*x*): *x *≤ *x*_0_} and ∑{*p*(*x*): *x *≥ *x*_0_}. For a continuous pdf the two areas are ∫−∞x0p(x)dx
 MathType@MTEF@5@5@+=feaafiart1ev1aaatCvAUfKttLearuWrP9MDH5MBPbIqV92AaeXatLxBI9gBaebbnrfifHhDYfgasaacH8akY=wiFfYdH8Gipec8Eeeu0xXdbba9frFj0=OqFfea0dXdd9vqai=hGuQ8kuc9pgc9s8qqaq=dirpe0xb9q8qiLsFr0=vr0=vr0dc8meaabaqaciaacaGaaeqabaqabeGadaaakeaadaWdXaqaaiabdchaWjabcIcaOiabdIha4jabcMcaPiabdsgaKjabdIha4bWcbaGaeyOeI0IaeyOhIukabaGaemiEaG3aaSbaaWqaaiabicdaWaqabaaaniabgUIiYdaaaa@3B25@ and ∫x0∞p(x)dx
 MathType@MTEF@5@5@+=feaafiart1ev1aaatCvAUfKttLearuWrP9MDH5MBPbIqV92AaeXatLxBI9gBaebbnrfifHhDYfgasaacH8akY=wiFfYdH8Gipec8Eeeu0xXdbba9frFj0=OqFfea0dXdd9vqai=hGuQ8kuc9pgc9s8qqaq=dirpe0xb9q8qiLsFr0=vr0=vr0dc8meaabaqaciaacaGaaeqabaqabeGadaaakeaadaWdXaqaaiabdchaWjabcIcaOiabdIha4jabcMcaPiabdsgaKjabdIha4bWcbaGaemiEaG3aaSbaaWqaaiabicdaWaqabaaaleaacqGHEisPa0Gaey4kIipaaaa@3A43@. Note that both **a **and **b **include *p*(*x*_0_). This is by design, as it yields a meaningful result for any *x*_0 _and distribution *p*(*x*). In the extreme low-variance limit, for example, if the *p*(*x*) is an impulse *δ*(*c*), then A
 MathType@MTEF@5@5@+=feaafiart1ev1aaatCvAUfKttLearuWrP9MDH5MBPbIqV92AaeXatLxBI9gBamrtHrhAL1wy0L2yHvtyaeHbnfgDOvwBHrxAJfwnaebbnrfifHhDYfgasaacH8akY=wiFfYdH8Gipec8Eeeu0xXdbba9frFj0=OqFfea0dXdd9vqai=hGuQ8kuc9pgc9s8qqaq=dirpe0xb9q8qiLsFr0=vr0=vr0dc8meaabaqaciaacaGaaeqabaWaaeGaeaaakeaaimaacqWFaeFqaaa@3821@(*x*) = 0 for all *x *except *x *= *c*, where A
 MathType@MTEF@5@5@+=feaafiart1ev1aaatCvAUfKttLearuWrP9MDH5MBPbIqV92AaeXatLxBI9gBamrtHrhAL1wy0L2yHvtyaeHbnfgDOvwBHrxAJfwnaebbnrfifHhDYfgasaacH8akY=wiFfYdH8Gipec8Eeeu0xXdbba9frFj0=OqFfea0dXdd9vqai=hGuQ8kuc9pgc9s8qqaq=dirpe0xb9q8qiLsFr0=vr0=vr0dc8meaabaqaciaacaGaaeqabaWaaeGaeaaakeaaimaacqWFaeFqaaa@3821@(*x*) = 1. The accuracy is thus always defined, and ranges from 0, when *x*_0 _lies completely outside the distribution, to 1, when *x*_0 _is the median of the distribution.

### Supplementary Figures, Tables, and expressions

Figures, tables and expressions with labeled beginning with 'S' are in Additional file 1.

## Results

Some interesting developmental splicing changes are evident simply form inspection of table S1. For example, the adult brain expresses a complement of structures that is very distinct from the fetal-brain population. A large portion of the difference is attributable to a developmental switch from insertion of segment **26 **to **25C **between constitutive exons 25B and 27. A broad array of other changes occur as well, most due to changes in splicing correlations. For example, the fetal brain expresses a significantly more diverse population of transcripts (from a much less diverse population of expressing cell types) than the adult brain, even though the marginal entropies of the alternative loci are highly similar and predict populations of equivalent diversity. See reference 8 for a detailed discussion of these matters.

### Clock plots

**Figure 3:**
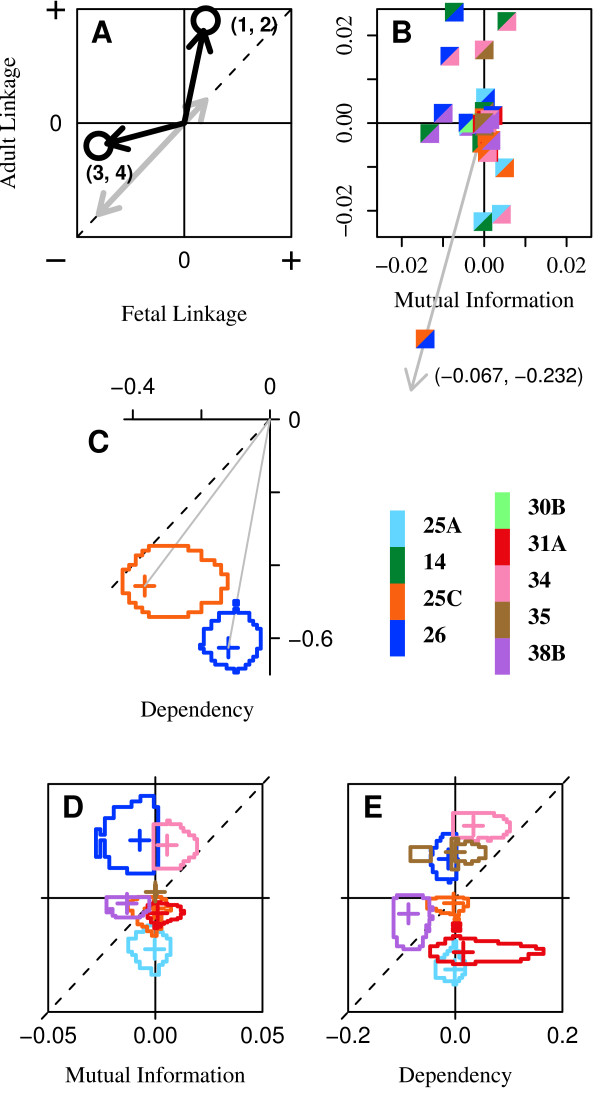
**Clock plots**. **A**, Illustrative example. Each circle represents a pair of alternative loci (e.g. loci '1' and '2'). The gray vectors depict splicing linkage that is present but does not change with development. **B**, Mutual information clock plot for all 36 pairs of the nine loci in the fetal and adult cDNA populations. Pairs of loci are identified in the plot symbols by colors defined in the key. The reference configuration is the insertion for every locus. **C**, Dependency clock plot for the single pair of loci (**25C, 26**). orange: *D*(**25C|26**); blue: *D*(**26|25C**). **D**, Mutual information clock plot for the eight pairs involving locus **14**: *I*(*x*, **14**), ∀*x *≠ **14**. **E**, Dependencies, D(x|**14**), of the same pairs.

Figure 3A plots splicing linkage between a pair of loci at one developmental stage *versus *another to display the developmental change in linkage. We call this a 'clock plot:' Each point is a vector whose magnitude measures the overall splicing linkage between the two loci and whose direction (displacement from the diagonal) indicates developmental regulation of linkage. Splicing may be developmentally regulated at both loci, but if they are regulated independently the plot point will fall on the origin, no matter how great the changes in splicing. If two loci are linked, but their *linkage *does not change with development, the point will lie away from the origin but on the diagonal. Thus, one pair of loci (1 and 2) shows a slight positive correlation between the reference configurations at the early stage, but this correlation increases greatly with development. The second pair also undergoes a developmental change in linkage, but in this case the loci become less correlated at the later stage. Linkage in this case is configuration-specific mutual information, which may be positive or negative. Plotting the configuration dependence allows us to see a reversal in the direction of correlation – manifest as a reflection about one axis – that may occur even in the absence of a change in mutual information.

Figure 3B is a clock plot displaying all 36 pairs of the nine alternative splice loci in the CACNA1G gene in fetal and adult human brain (data are in Table S1). The points are dispersed primarily along the adult axis, indicating a general developmental increase in splicing linkage among most pairs of loci, an interesting exception being those that involve locus **38B **(violet). Splicing at one pair of loci in particular, **25C **and **26**, is highly linked, with insertion at one locus favoring deletion at the other in both stages of development, but much more strongly so in the adult than in the fetal brain. Several loci show considerable pair-wise splicing linkages with multiple other loci. We note that domains that correlate structurally in this way are good candidates for some kind of functional relationship, and multiple pair-wise splicing linkages to a single locus, as seen here, may reflect either simple pair-wise functional interactions or a higher-order interrelationship. We explore the latter possibility in the next section.

Figure 3C, plots the (configuration-specific) *dependency *of **25C **on **26 **(orange) and that of **26 **on **25C **(blue). The dependency measures the extent to which splicing at one locus predicts splicing at the other. Unlike mutual information, the dependency is an asymmetric function of the two loci, and may reveal relationships that are less apparent with mutual information. In this case the strong developmental change in linkage is manifest more as a change in dependency of **26 **on **25C **rather than the other way around, as indicated by the much greater displacement of the blue point from the diagonal. This reflects a greater discrepancy in entropy of those two loci in the fetal population than in the adult (Table S3).

The statistical uncertainty, indicated by the dispersion around each data point in Figure 3C–E, for example, is obtained from 1000 simulated populations for each tissue, sampled by Monte Carlo from the empirical Bayes estimate (described below) of the distribution of splice variants in each tissue. For every pair of loci in each tissue, the mutual information (or dependency) values were binned into a histogram. Because splicing in the two tissues may safely be considered independent, the two-dimensional joint distribution for a pair of loci is the Cartesian cross product of the resulting bin-counts vectors from the two tissues. The error ring encloses the 95% most-probable values in this case.

Figures 3D and 3E allow comparison of mutual information and dependency for the same pairs of loci, in this case all pairs involving locus **14**. The data represent 1000 populations sampled from the empirical Bayes estimate, and error rings enclose the 95% most-proabable values for each pair of loci.

### Pairwise linkage grids

**Figure 4:**
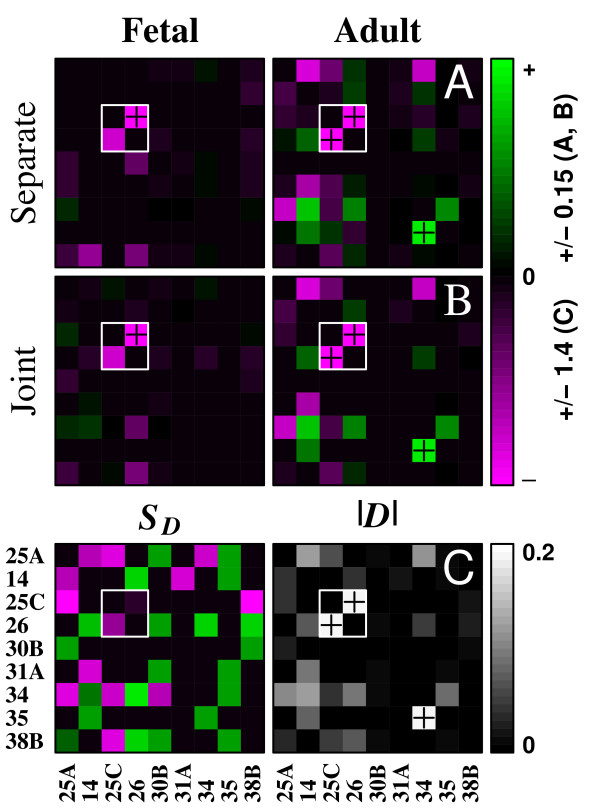
**Linkage grids**. **A, B**. Dependency, *D*(*i*|*j*), of splicing at one locus *i *on a second locus *j *is plotted for all pairs of loci in the fetal and adult cDNA populations. Independent loci *j *are on the abscissa. The layout is the same for all grids. Only dependencies at or above 99% significance are displayed. Statistical significance was determined for the two tissue samples either separately (A) of jointly (B). For a given tissue, the same values are plotted in A and B when the linkage is significant in both. Note that a linkage may be significant in one tissue but not both *or vice versa*. **C**. The linkage change index, *S*_*D *_(left), measures the extent of *change *in linkage at a pair of loci in a single parameter. Only changes significant at or above 99% are shown. The right panel plots the magnitude of the linkage vector, |*D*|, which gives an indication of the overall level of linkge at each pair of loci. The color scale ranges from -0/15 to +0.15 on the top four grids and from -1.4 to +1.4 on the bottom left. Some cell values fall outside the indicated range in order to improve the dynamic range overall. Plus signs (+) indicate these saturated cells.

Figure 4A displays linkage grids, showing the splicing dependency of all pairs of loci in a population that are statistically significant at the level of 99%. The rows give the dependent variables and columns the independent variables. In this way we may compare in adjacent grids the extent of splicing linkage in two populations for all pairs of loci exceeding a desired significance level. It is readily apparent in these plots that brain maturation entails the appearance or strengthening of a considerable number of pair-wise correlations.

Figure 4B plots the dependency values for those loci showing a statistically significant *developmental change *in linkage. Thus, whereas in Figure 4A the significance was determined separately for each tissue, in Figure 4B it was determined for both jointly. Note that while loci **25C **and **26 **show a high degree of negative dependency in both tissues (*c.f*. the red cells in the small white-bordered box within each grid of Figure 4A), the dependency of **25C **on **26 **does not change significantly with development, whereas the reverse dependency increases somewhat. This reflects the different positions of the two points in Figure 3C, where the orange ring touches the diagonal.

We have introduced the *linkage change index*, *S*_*D*_, to quantify these changes (*c.f*. methods). Figure 4C plots *S*_*D *_and |*D*| for those pairs of loci with nonzero *S*_*D *_at 99% significance or greater. The left grid shows the dynamic range of directional changes, while the right shows the overall magnitudes of the linkages involved. An interesting point of comparison is the values within the white-bordered box representing loci **25C **and **26**. These cells are quite dim within the *S*_*D *_grid, whereas they are bright in the other grids. This shows that, while there is strong splicing dependency between these two loci at both stages, and there is a statistically significant change in linkage with development, the extent of change is actually not very great in comparison to that at other pairs of loci. As we noted above, though, the dependency of **25C **on **26 **undergoes a greater developmental change than the reverse dependency; this is more apparent in the plot of *S*_*D *_than in the other plots. Other significant changes are much more obvious here as well.

### Spliceprints

Up to this point we have considered splicing linkage between pairs of loci. It is possible for splicing to involve correlations of higher order. For example a segment may be deleted at locus 1 only if segments are inserted at both loci 2 and 3. This situation necessarily entails pair-wise correlations between loci 1 and 2 as well as 1 and 3, but a three-way linkage is more intricate than a collection of disjoint pair-wise linkages.

Statistical interactions between splicing events at separate sites may be expected to relate directly to functional interactions between the alternatively spliced domains. It is only through this mechanism that alternative splicing may exert control over the function of the expressed proteins. The nature of physico-chemical interaction between domains in a protein is extremely complex, generally unpredictable, and not limited to "nearest-neighbor" effects. This means that functional interactions may be *expected *between arbitrarily many domains, and that functional effects of a configuration change may be highly nonlinear, depending on the complement of variable domains at other sites in the same protein. We have demonstrated highly nonlinear (non-additive) functional effects, exactly of this nature, in the CACNA1G ion channel electrophysiology [8]. Thus we should not be surprised to find higher-order splicing interactions, reflecting control of domain combinations at multiple loci, and we do not expect this phenomenon to be limited to the present system.

**Figure 5:**
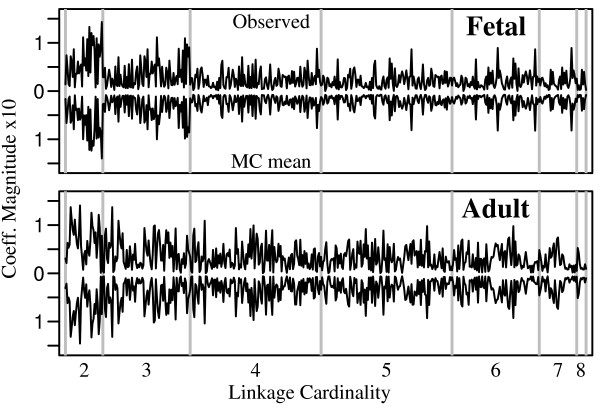
**Spliceprints**. Log-linear coefficient magnitudes are plotted for all subsets of more than one locus. The vertical scale is the same for all traces. Within a cardinality, *k*, the sequence of coefficients is determined by listing the 9 loci from left to right as in Figure 4 (abscissa), and choosing groups of loci from left-most to right-most as follows: for loci A = **25A**, B = **14**, etc., cardinality-3 coefficients occur in the order ABC, ABD, ACD, BCD, ABE,..., PHI, GHI.

We model multi-site alternative splicing with a log-linear model [25] to quantify higher-order linkages. Figure 5 displays the amplitudes of the log-linear coefficients from two developmental stages, for all terms of order 1 or higher in the saturated model. The contributing loci are not identified here, but terms for subsets of the same cardinality are grouped together between vertical rules. In each plot, values above the zero line are coefficients derived from the Empirical Bayes estimate of the experimental population. For comparison, traces below the midline plot mean values from 1000 Monte Carlo populations. Because only the magnitudes are plotted, the ordinate values increase with distance from zero both upward and downward. Differential splicing regulation in the fetal and adult brain appears as different patterns in the upper and lower boxes. Notice that independent splicing gives coefficients that lie on the zero line, and that the more compressed fetal pattern indicates a lower level of splicing correlations, especially at higher orders. Although we focus only on the magnitudes of the log-linear coefficients in the present work, a wealth of information is present in their signs, which would admit a multidimensional extension of the clock plot analysis. Also, of course, the spliceprint is not limited to log-linear coefficients, and the coefficients may be presented in any desired order on the abscissa.

### Minimal-linkage models

We may wish to identify the smallest set of interactions that can account for the data, within bounds of statistical significance. It may be surprising, for example, that some genes display nearly independent splicing at multiple sites, even in rather complicated tissues [8,17,19]. In such cases one or two pair-wise interactions may account for any deviations from independence. We may identify those by first ranking the pairs in order of decreasing mutual information, then define a hierarchical model with only the most highly correlated first-order interaction terms. A least-squares fit to this model gives coefficients for only those interactions. We then ask whether this represents a possible parent distribution for the observed population.

A simple way to do this is to sample Monte Carlo populations from the fitted distribution, with the same number of transcripts, *N*, as the experimental population. For each MC population we then calculate its likelihood of arising from a reference distribution, *ρ*, for example:

ρ=N!∏v=0NT−1pvnv/nv!     (4)
 MathType@MTEF@5@5@+=feaafiart1ev1aaatCvAUfKttLearuWrP9MDH5MBPbIqV92AaeXatLxBI9gBaebbnrfifHhDYfgasaacH8akY=wiFfYdH8Gipec8Eeeu0xXdbba9frFj0=OqFfea0dXdd9vqai=hGuQ8kuc9pgc9s8qqaq=dirpe0xb9q8qiLsFr0=vr0=vr0dc8meaabaqaciaacaGaaeqabaqabeGadaaakeaaiiGacqWFbpGCcqGH9aqpcqWGobGtcqGGHaqidaqeWbqaaiabdchaWnaaDaaaleaacqWG2bGDaeaacqWGUbGBdaWgaaadbaGaemODayhabeaaaaGccqGGVaWlcqWGUbGBdaWgaaWcbaGaemODayhabeaakiabcgcaHaWcbaGaemODayNaeyypa0JaeGimaadabaGaemOta40aaSbaaWqaaiabdsfaubqabaWccqGHsislcqaIXaqma0Gaey4dIunakiaaxMaacaWLjaWaaeWaaeaacqaI0aanaiaawIcacaGLPaaaaaa@4A16@

This is a multinomial distribution of *N*_*T *_splice variant classes, *v*, each with expected probability *p*_*v *_and abundance *n*_*v *_in the sample: ∑*n*_*v *_= *N*. The reference distribution is in fact arbitrary: we are not interested in the exact likelihood of our data; rather, we wish to find a model that generates populations of likelihood similar to the data with a given reference distribution. The fraction of MC likelihoods not exceeding the observed value measures the evidence against the model. This approach is an approximation to the method of posterior predictive assessment of model fit of Gelman *et al*. [26]. Expression (4) constitutes their statistic *T*. We seek a model with sufficient departure from independence to be consistent with the observed data.

**Figure 6:**
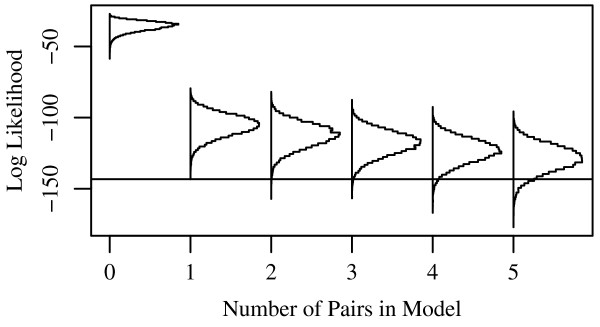
**Posterior predictive assessment of minimal model fit**. A simple model may account for the observed distribution within admissible error limits, but it may cause one to overlook important effects in a network of interactions. Histograms of log-likelihood (assuming independent splicing) are presented, each for 1000 Monte Carlo populations, sampled from a series of models incorporating an increasing number of pairwise correlations. See text for details.

Figure 6 illustrates this method with the adult data. It shows log-likelihood histograms for six Monte Carlo ensembles of 1000 populations each. The reference distribution is the independent-splicing expectation [equation (1)] of the experimental population. The horizontal rule at -143 shows the log-likelihood of the observed population. The first ensemble (left-most histogram) was sampled from the reference distribution, and shows that independent splicing is inconsistent with the experimental data. The reference distribution gives the 'cardinality-1' log-linear model, *log p*(*C*_123...*k*_) = *u *+ ∑_*j*_*u*_*j*_(*C*_*j*_), with all independence terms and no interactions. The next histogram is obtained by adding a single interaction to this model: *u*_34_(*C*_34_), the pair-wise interaction between loci **25C **and **26**, identified by mutual information as the most highly correlated segments. Each subsequent ensemble is obtained from the previous by adding the next most-correlated pair. A minimum of five pair-wise linkages is thus required to account for the observed splicing correlations by a stochastic mechanism within bounds of 95% confidence.

### Saturated models

Peptide domains that interact functionally are likely to exhibit statistical correlations reflecting enrichment of productive interactions or suppression of detrimental ones. Figures 1 and 2 show that many individual loci participate in pair-wise interactions with multiple other loci. Where these reflect functional interactions, we anticipate two important consequences, due to the fact that they occur within a close-packed, folded protein: (i) they are likely to be *transitive *in nature – *e.g*., if loci A and B interact and loci B and C do, we expect that A and C will as well. Furthermore, we should expect that splicing at C will have effects that depend on A and B *together, i. e*. (ii) a set of loci may influence protein function as an integrated *ensemble*, rather than a collection of functionally separable modules. It then follows that their splicing regulation will exhibit mutual, higher-order interdependencies. Unsaturated models cannot capture these correlations accurately: A low-order model, as in Figure 6 for example, is from this perspective an oversimplification, emphasizing a few low-order interactions at the expense of a wealth of information in the higher orders. Though we correctly identify the most highly correlated domain pairs, this gives no clue how pairs may link up within a molecule, *i.e*., evidence for ensembles of functionally interacting protein domains.

**Figure 7:**
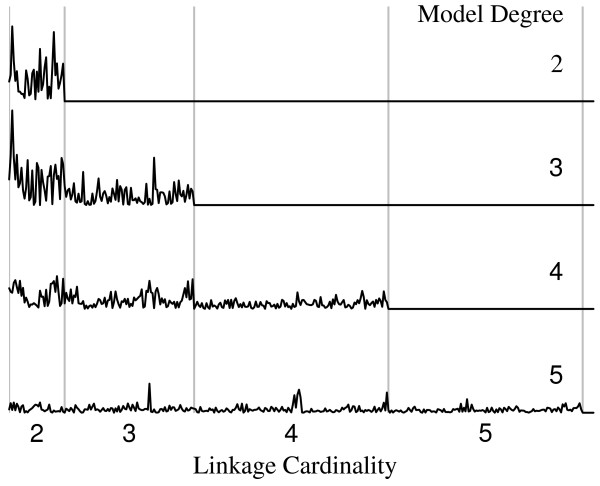
**Spliceprints of successively higher-order hierarchical models fit to the same data**. Excluding high-order effects from the model changes the *relative *magnitudes of the remaining coefficients, thus misrepresenting the lower-order interactions. The vertical scale is the same for all plots.

Figure 7 shows the distribution of amplitudes among the first 4 orders of interaction terms (cardinality *k *= 2,...,5) from fits of four hierarchical models to the same data (adult population). Each panel plots coefficients from a model that includes all terms of cardinality *k *and lower, but none higher. When excluded from the model, high-order interactions are 'absorbed' into lower-order terms. Notice, for example, the redistribution of *relative *amplitudes within the cardinality-3 coefficients as higher-order terms are included in the model. This occurs as weight from triplets in higher-order linkage groups is shifted to their higher-order coefficients when they are made available in the model. Since we wish to compare coefficients estimated under identical models from parallel data sets, we use a saturated model to avoid confounding the low-order terms with higher-order effects. A nonzero coefficient for a set of loci then indicates a mutual splicing dependency among all loci in the set, in excess of any lower-order interactions that may be present among component subsets, and larger magnitudes reflect stronger correlations.

### Empirical Bayes methodology

We are not interested in the precise values of the coefficients as much as the relative amplitudes of the same coefficients from two different populations, as compared side-to-side in Figure 5, for example. From this we may discern statistically significant developmental shifts in splicing linkages. This requires that we estimate the variance of the log-linear coefficients. These may be obtained from a saturated model fit to Monte Carlo populations sampled from an estimate of the parent distribution.

The simplest such estimate is the observed distribution itself (the bootstrap). While this makes no assumptions about the underlying mechanism, it assigns zero probability to the unobserved splice forms, which is obviously unreasonable. Increasing the experimental sample size, even by an order of magnitude, may not make the bootstrap applicable if the number of alternatively spliced loci is even moderately large (Figure S2): the probability space expands geometrically with the number of variables, so unless the sample is vastly larger than the number of classes the table of observed frequencies will contain a large number of zeros (empty cells). This is exaggerated when splicing at any locus is strongly biased, as is common (*e.g*. Figure S5). Transcripts that combine rare splice configurations at multiple loci thus have a very low expectation, though we cannot assume that any empty cells would persist if we continued data collection indefinitely.

The empirical Bayes approach [27] enables an estimate of the parent distribution with plausible nonzero probabilities for the unobserved classes. This estimate (the *posterior *distribution) incorporates the observed distribution (the *likelihood*) with our current understanding of the underlying process (the *prior *distribution). We have found the 'pseudo-Bayes' estimator of Bishop *et al*. [25], a linear shrinkage estimator chosen for its simplicity, to be entirely adequate. Improvements may be made to the estimator – with nonlinear shrinkage, for example, but typically at the expense of added complexity. Our models do exhibit sensitivity to the choice of prior distribution, however, because of the sparse representation of splice forms in the experimental data. We present a thorough examination of different priors in the Supplementary Information. Because splicing at separate loci is approximately independent (Figure S4), equation (1) provides an excellent prior: the *tissue-specific independent marginals *prior. In this work we primarily use the *averaged-marginals *variant of this prior, obtained by averaging the fetal and adult expected frequencies.

The empirical Bayes methodology is open to the criticism that including experimental results in the prior may lead to duplicate use of evidence and subsequent underestimation of uncertainty. Purportedly 'uninformative' priors inadvertently introduce their own errors, however, mainly by forcing untenable splice correlations into the estimator (*c.f*. Supplementary Information; also Figure 9). By making judicious use of the observed marginal frequencies in the prior we minimize this effect, and keep the focus of inference on the interactions. The present method has a major advantage in providing a simple comparison of two developmental stages by linkage order, with low computational burden.

**Figure 8:**
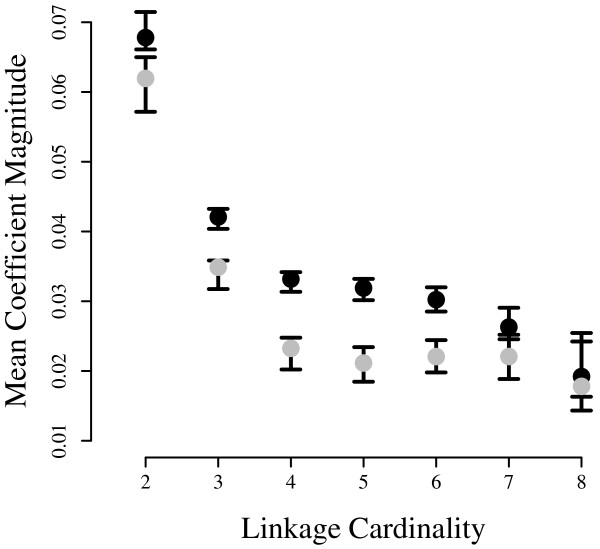
**Cardinality-averaged log-linear coefficients**. All coefficients of the same degree are presented as a single average magnitude. 1000 Monte Carlo populations were sampled from the empirical Bayes posterior obtained with the 'averaged-marginals' prior and fetal (gray) or adult (black) likelihood. Error bars delimit the 2.5 – 97.5% interquantile range for each distribution.

**Figure 9:**
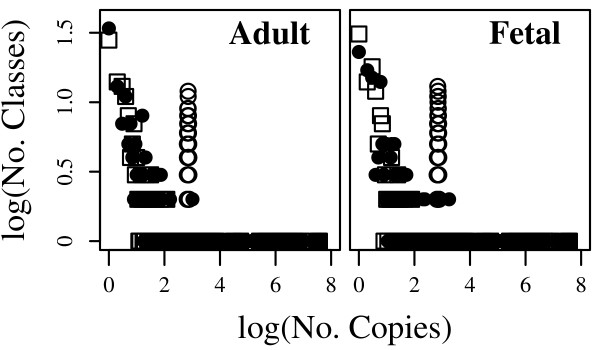
**Frequency-of-frequency plots**. Monte Carlo populations of 10^8 ^transcripts each were sampled from the empirical Bayes estimate obtained with the fetal or adult likelihood and either the tissue-specific independent marginals (**dots**), averaged-marginals (**squares**), or uniform (**circles**) prior. The uniform prior gives an idiosyncratic L-shaped profile with an abrupt lower copy-number limit (open circles). This reflects the implicit exchangeability of the unobserved classes: all have the same low probability, but because the majority of forms are not observed, their cumulative probability in the estimator is large. This is one example of how the uniform prior (or any prior obtained by a small constant correction to the observed frequencies), although 'uninformative', is overly simplistic, and leads to artifacts.

### Developmental changes in higher-order linkages

Figure 8 presents a statistical summary of Figure 5, obtained with the averaged-marginals prior. The adult population displays increased higher-order correlations compared to the fetal for groups of up to at least 6 loci. This agrees with the mutual information results (*e.g*. Figure 3B), with an interesting additional feature: the fetal and adult profiles are most divergent at cardinalities 4 and 5 with the gap closing toward cardinality 2. This shows that much of the difference in mutual information between the two tissues derives from extensive splicing correlations involving sets of considerably more than two loci, whereas the extent of isolated pair-wise interactions is more nearly comparable in the two tissues. Elsewhere we demonstrate that these higher-order splicing correlations correspond to non-additive functional interactions among multiple domains in the expressed ion channel protein [8]. The spliceprint provides compelling evidence that entire ensembles of domain configurations are selected in concert during processing of the RNA transcript, and that this process becomes more restrictive in the course of development. These loci are spread out over a large portion of a large transcript, separated by ten or more introns in some cases. Fededa et al [28] present one mechanism of long-range coordination of alternative splicing, and others most likely will be found.

### The SGT is a transcriptome

Though we have used actual SGT data and a detailed multivariate stochastic splicing model to present analyses of splicing correlations, our focus is on the methods themselves, which apply regardless of the dataset or details of the splicing model. Only with a realistic splicing model, of course, can we make reliable statistical inferences. We present a detailed rationale for our model as a supplement [see Additional file 1]. This model may be further refined, for example through computational methods of variable selection (Dahinden, Parmigiani, Emerick, and Bühlmann, manuscript in preparation) or collection of additional data from different sources. A realistic splicing model allows us to investigate the single-gene transcriptome with established methods of transcriptome analysis. Figure 9 presents one such approach.

The transcriptome is a highly complex assortment of gene products, but it exhibits a remarkably stable expression pattern. Only a few genes are expressed at a high level, while most genes are represented by only a few copies. It is not clear that this pattern should persist at the single-gene level. Different physiological inputs would affect the profile at different levels, so those aspects of gene-network topology that conspire to shape the aggregate gene expression profile, for example, may or may not be relevant to the selection of splice isoforms from a single gene in separate cells or tissues. Nonetheless, the basic characteristics of the transcriptome profile are also present in its elemental building block, the SGT. Figure 9 shows 'frequency-of-frequencies' plots for simulated SGTs sampled from the tissue-specific independent-marginals distribution (filled circles). The reverse-J pattern, like those obtained in genome-wide expression profiles assayed by SAGE [29], reflects the complexity of both the transcript inventory and the tissue physiology in which this gene is expressed. The identical profile was obtained with the averaged-marginals estimator (squares), which places a lower reliance on the observed marginal splicing frequencies in either tissue. (A uniform prior distribution (open circles), which we have shown to be inappropriate for a wide range of reasons yields an idiosyncratic L-shaped profile with an abrupt lower copy-number limit).

This analysis shows that the SGT exhibits a complex expression profile similar to that of the complete transcriptome, but that this is true whether or not splicing correlations are present at observed levels. Splicing correlations may represent a level of organization unique to the single-gene level. Alternatively, of course, analysis of correlated gene expression in the whole-genome transcriptome may reveal comparably interesting behavior.

## Discussion

The human genome supports in the neighborhood of 23,000 protein coding genes [30], very similar to the number found in genomes of vastly simpler organisms, such as *C. elegans *and *Drosophila *[31,32]. To account for the increase in human phenotypic richness, therefore, the number of structural genes is not as important a factor as the way in which genes are used. Variations in gene expression levels, changes in the timing of expression, evolutionary adaptations that rearrange gene interactions as well as evolution of the coding sequence, and increased post-transcriptional modification of primary transcripts to diversify the products of single genes all play a role [9,33].

Here we present tools to evaluate and visualize complex patterns of transcriptome variation, illustrated on populations of full-length cDNA splice variants from CACNA1G, the gene encoding the human Ca_*v*_3.1 T-type calcium-channel *α*_1 _subunit. In the course of brain maturation the transcriptome of this gene undergoes a transformation that would be largely invisible to a study of gene expression levels or a microarray- or EST-based splicing survey. The changes appear only in the complete structures of full-length transcripts, as alterations in splicing correlations at separate loci within the same molecule. A standard analysis of pairwise correlations, while illuminating, is incomplete in an important way. Compared to the fetal transcriptome, the adult displays a marked increase in mutual information between many pairs of loci (Figure 4). The multivariate analysis, however, reveals two components of this increase: a modest elevation in disjoint pairwise linkages and a substantial increase in higher-order correlations that include linked pairs as a subset. Overall, splicing in the adult is far more restrictive than fetal splicing. This occurs at the same time as the range of cell types in which this gene is being expressed is diversifying, not constricting. This is consistent with the notion that splicing may need to be more stringently specified in the more intricate 'wiring' of the mature brain [8].

It is the grounding principle of this work, therefore, that splicing correlations will generally reflect functional interactions, and that these are likely to involve multiple domains. Splicing of physically linked domains should be co-regulated to inhibit detrimental interactions as well as to enhance beneficial ones. This relates directly to the complexity of the processes that regulate selection of alternative domains, the most important factor being whether the domains are modular or functionally interactive.

*Modular domains *may be shuttled in or out with predictable effects, independent of splicing at other loci. They may be used to conjoin functional activities – post-synaptic targeting with fast activation gating in an ion channel, for example. *Interactive domains*, in contrast, express a shared functional effect that exists only in the context of the ensemble. A specific effect cannot be independently defined for a single interactive domain: reconfiguring one such domain 'reinterprets' the functional influences of the others.

That is, the molecular phenotype may be expressed as a linear combination of the effects of modular domains, but not so for interactive ones. As an example, deleting segment **38B **of the Ca_*v*_3.1 calcium channel decreases the window current magnitude when **25C **is present, but increases it when **26 **is present, and has no effect when both are absent; furthermore, it does not effect gating rates, except when **14, 25C **and **26 **were all absent, whereupon it speeds inactivation [8]. Whether domains interact functionally depends on the domains, and modular and interactive qualities are not mutually exclusive.

The number of alternative molecular phenotypes is the same whether the loci are modular or interactive. In the former case, however, any given state is decomposable into identifiable subsets of phenotypes, whereas in the latter it is not. Functional interactions admit the possibility of introducing completely unpredictable, *qualitatively *novel behavior simply by reconfiguring an existing set of domains. In the course of evolution, the simple addition of a new variable domain may reinterpret the phenotypes of existing splicing patterns, enabling a rapid expansion of functional alternatives from the ancestral gene.

Non-additive functional interactions may have various causes. Inserting one domain may simply block access to a binding site for a second domain, for example. Another possibility is an 'allosteric' type of interaction where electrical or conformational changes communicate through the protein interior. The consequences of such interactions may become even more complex when other genes are alternatively spliced in multiple ways. Current estimates of the number of alternatively spliced genes in humans range to ~76% of known genes [34], with an average of 3.9 splicing isoforms per gene [1]. Furthermore, the frequency of alternative splicing is elevated in genes that mediate cell signaling and metabolic networks [34], increasing the likelihood of nonlinear, and largely unpredictable interactions *between genes *with alternatively spliced products that communicate through such networks. Strong intergenic interactions are of course normal where proteins contact physically, as subunits of a multi-enzyme complex, or in a multi-subunit ion channel. There are 22–25 such genes for voltage-dependent calcium channels, all of which may be alternatively spliced. These may assemble in up to 840 stoichiometric complexes, excompassing as many as 20 variable sites each. Physiological channels may arise from as many as ~9 × 10^8 ^transcript combinations. This is an enormous space of possibilities, just for calcium channels, that can be exploited in the refinement of neuronal networks.

We may expect splicing correlations to cross gene boundaries in such cases, though direct physical contact may not even be necessary in general [35]. Splicing linkage analyses in high-throughput transcriptomics may provide a valuable compliment to direct peptide interaction studies, such as yeast two-hybrid, to reveal functional interactions that do not require strong physical contacts. It is interesting, in this light, that the notion of a reconfigurable 'interactome' [36] extends to variable domains within the protein interior.

The unpredictable consequences of functional interactions are amplified through ambiguity in the determinants of alternative splicing, which are not fully specified in the gene sequence. Complex mammalian genes support an intrinsic uncertainty in the structure of the expressed protein which is reduced epigenetically, through information residing outside the gene, within networks of *trans *regulatory factors, for example [37-39]. Thus, very rare splice configurations may be produced under most conditions. Though any particular one may have a low probability, there is always a chance that a new form may arise, producing a protein that functions, albeit in an unusual way. A low level of such 'noise' may in fact be useful to a cell in a complex, unpredictable local environment. This certainly describes the mammalian brain, where humans have far outpaced other primates in the evolutionary divergence of phenotype. In keeping with this, the brain expresses a disproportionate diversity of alternative splicing, compared to other tissues [40].

In the context of expanding complexity in alternative splicing, interactions between variable domains therefore present a challenge to the regulatory processes that select them. A set of *k *modular domains may be configured through *k *sequential yes/no choices. Functional interactions, however, force a single, non decomposable, selection from 2^*k *^alternatives. The regulatory complexity thus increases exponentially with the number of loci if they interact, but only linearly if they do not. This is the cost of diversifying the proteome through combinatorial splicing. It returns a significant payoff, however, because the exponential expansion of the regulatory load is compensated by an expansion of phenotypic potential on the same scale. We have noted [8] that this regulatory burden could be escaped if some mechanism were available for somatic selection during development, based on feedback from the expressed transcriptome.

## Conclusion

As full-length cDNA datasets become available the methods presented here will assist in defining the interaction landscape, revealing domain configurations that are selected in concert and providing insights into how domains within proteins interact functionally. Additionally, however, they should adapt well to studies of clustering in the transcription of genes (and parts of genes). A compelling application is to the class of RNA transcripts that do not encode proteins [41]. Though largely of unknown function, these ncRNAs comprise a large proportion of the transcriptome, representing roughly 50% of transcriptional units and covering 30 times more of the genome than the protein-coding mRNA, and they are elaborately processed (capped, polyadenylated and spliced – constitutively, alternatively and *trans-spliced*) [20]. They likely represent an important component of the intricate web of RNA factors involved in the regulation of gene expression [42], including regulated alternative splicing.

## Authors' contributions

ME conceived the study, collected the data, designed the algorithms, code, graphics, and novel indices, with input from WSA on the linkage change index, performed the analyses and wrote the paper, with significant supporting contributions from GP. GP suggested the Bayes/log-linear approach, and provided invaluable guidance, being especially receptive to "interesting" ideas. WSA provided funding and valuable consultation, and initiated work on the linkage change index. All three authors read and approve of the final manuscript.

## Supplementary Material

Additional File 1 A PDF file with a table listing the data used for these analyses (Table S1), a graphical scheme illustrating how modular and interactive splicing relate to splice configuration (Figure S1), and a detailed exposition of the empirical Bayesian approach, with an extensive analysis of sensitivity to the choice of prior distribution. Click here for file
