# Supplementary material for: Multivariate Analysis and Visualization of Splicing Correlations in Single-Gene Transcriptomes
Source: BMC Bioinformatics. 2007 Jan 18;8:16. doi: 10.1186/1471-2105-8-16 (PMC1785386; doi:10.1186/1471-2105-8-16)
Supplement: Additional File 1 — A PDF file with a table listing the data used for these analyses (Table S1), a graphical scheme illustrating how modular and interactive splicing relate to splice configuration (Figure S1), and a detailed exposition of the empirical Bayesian approach, with an extensive analysis of sensitivity to the choice of prior distribution. [file 1471-2105-8-16-S1.pdf]

Suppplement to Multivariate Analysis and Visualization of Splicing Correlations in Single-Gene Transcriptomes

Mark C. Emerick, Giovanni Parmigiani, William S. Agnew

September 14, 2006

Contents

1 Datasets **Supp. 2**

2 Illustration of modular and interactive domains **Supp. 3**

3 Empirical Bayes methodolgy **Supp. 4**

3.1 Choice of prior distribution . . . . Supp. 4

3.2 Assessing goodness of fit . . . . Supp. 10

Table S1

	Counts		Configuration								
Species	Fetal	Adult	**Δ25A**	**14**	**25C**	**26**	**30B**	**31A**	**34**	**35**	**38B**
17	1		○	○	○	○	●	○	○	○	●
25	9	36	○	○	○	○	●	●	○	○	●
33	1		○	○	○	●	○	○	○	○	●
49	1		○	○	○	●	●	○	○	○	●
57	64	11	○	○	○	●	●	●	○	○	●
88		2	○	○	●	○	●	●	○	○	○
89	4	250	○	○	●	○	●	●	○	○	●
93	1		○	○	●	○	●	●	●	○	●
137			○	●	○	○	○	●	○	○	●
145	1		○	●	○	○	●	○	○	○	●
153	33	27	○	●	○	○	●	●	○	○	●
157	5		○	●	○	○	●	●	●	○	●
169	3		○	●	○	●	○	●	○	○	●
177	1		○	●	○	●	●	○	○	○	●
184	10		○	●	○	●	●	●	○	○	○
185	111	66	○	●	○	●	●	●	○	○	●
189	5	15	○	●	○	●	●	●	●	○	●
209		10	○	●	●	○	●	○	○	○	●
216		11	○	●	●	○	●	●	○	○	○
217	8	289	○	●	●	○	●	●	○	○	●
221		4	○	●	●	○	●	●	●	○	●
223		4	○	●	●	○	●	●	●	●	●
249			○	●	●	●	●	●	○	○	●
313	8		●	○	○	●	●	●	○	○	●
409	8		●	●	○	○	●	●	○	○	●
441	13		●	●	○	●	●	●	○	○	●
473		22	●	●	●	○	●	●	○	○	●
477		11	●	●	●	○	●	●	●	○	●
Total:	287	758									

1 Datasets

We analyze a biological dataset described in Emerick *et al.* (2006). Human $Ca_v3.1$ structural variants in full-length single-gene libraries from adult and fetal whole brain. The species designation is the decimal equivalent of the bit-string representation of splice configurations on the transcript, in the order listed.

2 Illustration of modular and interactive domains

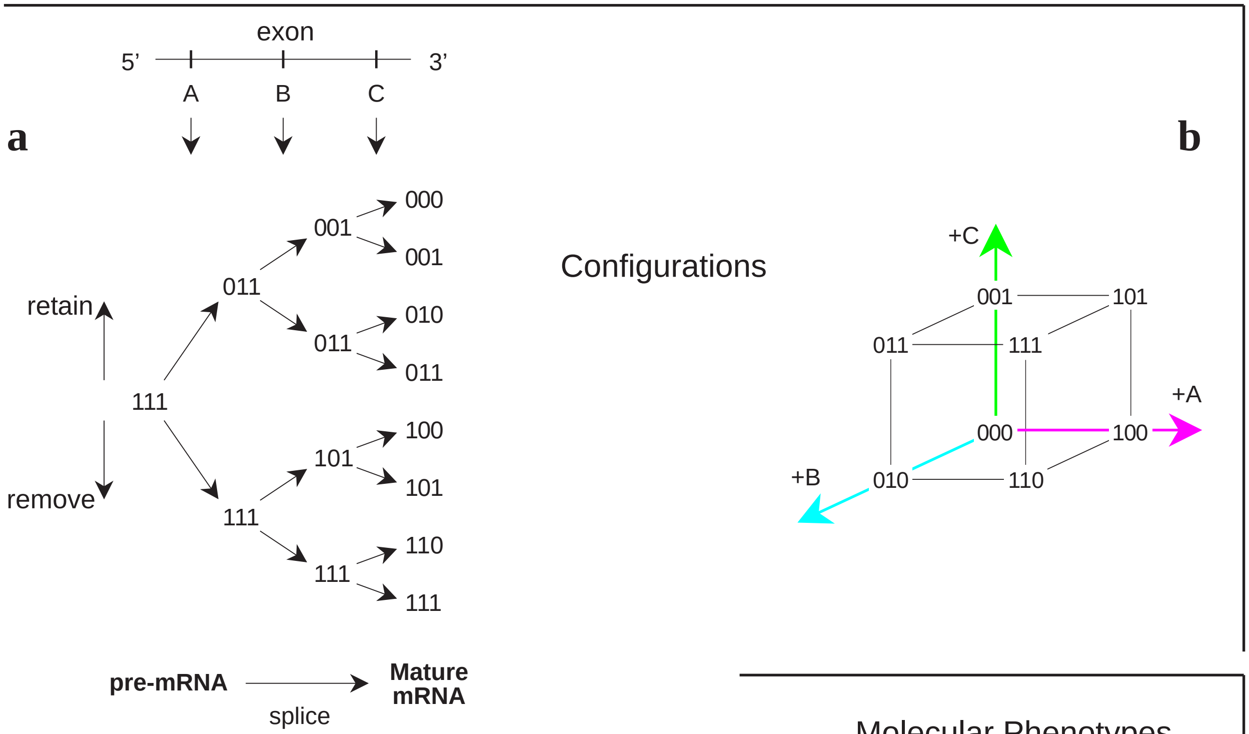

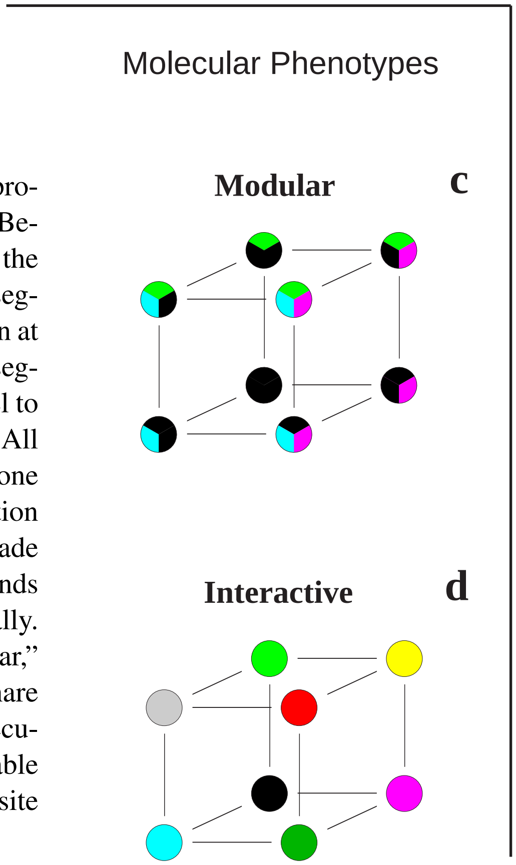

Figure S1: Splicing decision tree (**a**): In this model splicing proceeds sequentially from the 5' to the 3' end of the transcript. Beginning with the primary transcript (pre-mRNA) each node in the graph represents a decision to retain or remove a variable exon segment from the final message (mature mRNA). The configuration at each node is the final configuration if all downstream variable segments are retained. Configuration space (**b**): Movement parallel to one axis corresponds to a change in configuration at one locus. All nodes on one face of the cube share the same configuration at one locus, and all on the opposite face share the opposite configuration at that locus. The individual splicing decisions might not be made sequentially, but whether they can be made *independently* depends on whether the alternatively spliced domains interact functionally. If a domain does not interact with others, we say it is "modular," and all proteins with the same configuration at that locus share an identifiable phenotypic characteristic. The resulting molecular phenotypes, illustrated schematically in (**c**) are decomposable by domain. Interactive domains combine to produce a composite phenotype that is not decomposable in this way (**d**).

3 Empirical Bayes methodolgy

We estimate the frequency of splice variants in the parent distribution (the original tissue source) with the 'pseudo-Bayes' estimator, $\mathbf{p}^*$, of Bishop *et al.* (1975). This is a linear shrinkage estimator that takes a weighted average of the prior mean and the maximum likelihood estimate, shrinking the observed frequencies toward the prior, optimizing the weight to minimize the Euclidian distance between the estimate and the parent distribution (Carlin, 1996):

$$\mathbf{p}^* = (1-w)\mathbf{p} + w\boldsymbol{\lambda} \tag{S1a}$$

$$w = K/(N+K) \tag{S1b}$$

$$K = (N^2 - \sum_v x_v^2)/\sum_v (x_v - N\lambda_v)^2 \tag{S1c}$$

where N is the total number of transcripts in the cDNA library, x_v is the observed number of counts of each splice variant v, and λ_v is its prior probability. The likelihood, $\mathbf{p}$, is multinomial, $p(\mathbf{x}|\boldsymbol{\lambda}) \sim \prod \lambda_v^{x_v}$, [expression (4) in the paper], with integer parameters x_v. Our prior is Dirichlet: $p(\boldsymbol{\lambda}|K) \sim \prod \lambda_v^{\beta_v - 1}$, generally with non-integer parameters $\beta_v = K\lambda_v$. The posterior is therefore Dirichlet as well, with parameters $x_v + \beta_v$. We discuss extensively the choice of prior distribution.

3.1 Choice of prior distribution

Summary

If our goal were to model the observed population, the bootstrap would suffice. A realistic estimator should admit a small, nonzero probability for the unobserved classes, however. The two most common ways to do this are (i) a uniform prior, free of assumptions about the true distribution, and (ii) a prior obtained by adding a small constant to all observed frequencies and renormalizing. Both of these methods err in assigning equal probability to all unobserved classes. With a large number of such forms this becomes a large error, such that the observed classes are well modeled but the complete distribution is not. The unintended assumption, in effect, is that a high degree of splicing correlations conspire to lock out unobserved forms. A more likely scenario is that overall splicing is less restrictive and many of these forms would show up in larger samples.

We observe that the distribution of splice forms in both fetal and adult brain approximately follows the 'independent-marginals' expectation. Without justification for believing that unobserved forms would follow a different pattern, we may chose this distribution as our prior. This prior may be viewed as a smoothed modification of prior (ii), above, in which the probabilities of the unobserved classes taper off in a more realistic fashion, toward zero for forms that combine multiple low-frequency splice configurations. Because fetal splicing is more nearly independent than adult the fetal estimator gives more weight to its independence prior, and less weight to the actual data, than does the adult. A minor modification removes this effect: we reduce the amount of information about the tissue-specific marginal frequencies included in the prior by averaging the fetal and adult independence expectations into a single 'averaged-marginals' prior for both tissues. While omitting what we know about the developmental reversal at **25C** and **26**, this approach incorporates the information that splicing frequencies at the other 7 loci are preserved during development. The extent of shrinkage is approximately the same in the fetal and adult cases. The goodness of fit is similar for both and is not improved by arbitrarily reducing the shrinkage weight to favor the observed population.

Our criteria for assessing the prior distribution are that unobserved variants may have a small nonzero probability and that all observed frequencies should be among the more probable frequencies in the posterior distribution. A uniform prior is a simple option that allows modeling of the unobserved classes without imposing *a priori* differences between the unknown frequencies. This prior reproduces the observed behavior well (Figures S2A and S10, estimator 13) because the shrinkage gives little weight to the prior for either the fetal or adult tissue; w in equation (S1) is

Table S2: Loglinear model parameters for three priors.

	K		*w*	
	Fetal	Adult	Fetal	Adult
uniform	3.58	2.76	0.012	0.0036
tissue-specific marginals	146.76	32.80	0.338	0.0415
Averaged marginals	6.42	0.22	0.022	0.0003

0.004 for the adult and 0.012 for the fetal population (table S2). The posterior is thus the distribution of observed frequencies modified only slightly to give a nonzero probability to the unobserved classes. Shrinkage with this prior is preferable to the bootstrap in that it admits the possibility of sampling unobserved variants, but it does so in a rudimentary way, dismissing what we know about splicing at the separate loci. That is, some splice combinations are much more likely to occur than others, and it is just as unrealistic to suppose that all unobserved classes are equally likely (uniform prior) as it is to suppose that they are all impossible (bootstrap). In fact, simple independent assortment of multiple splice configurations, several with low probability, would produce many splice variants of extremely low probability while others would lie just beyond the detection limit at the current sample size.

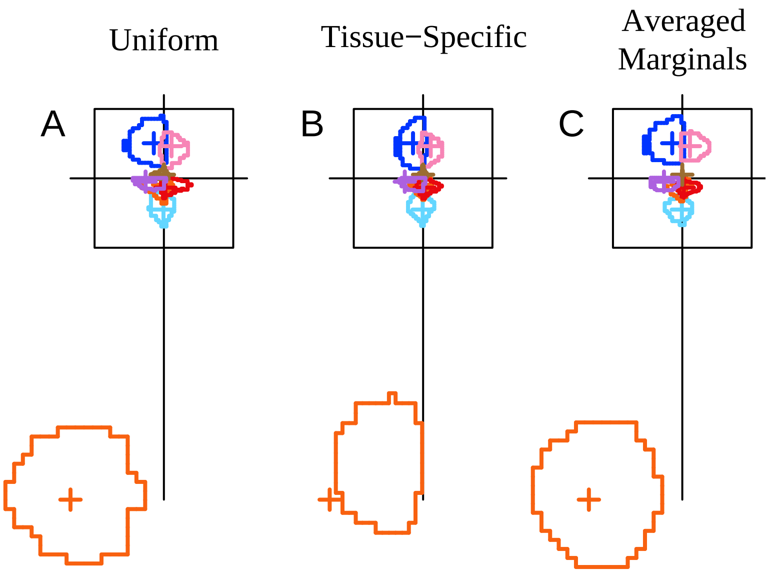

Figure S2: Mutual Information clock plots for three priors. Points within the box show pair-wise linkage of various loci to locus **14**. The orange plots differ in depicting linkage between loci **25C** and **26**. Error rings give 95% confidence limits

Figure S3A and B illustrate the latter point: if splicing at the separate loci were completely independent, a library of 5,000 cDNAs from either adult or fetal brain would yield only about 14% of the 512 possible splice variants, with diminishing returns for larger samples. The yield is about the same in both tissues, reflecting a similar distribution of marginal frequencies in both. When splicing is not independent fewer forms are obtained, and while the fetal population appears close to independence, the adult is not. Figure S3 shows the dependence of species richness on sample size for several other estimators. The bootstrap (panels C and D) models only the observed variants.

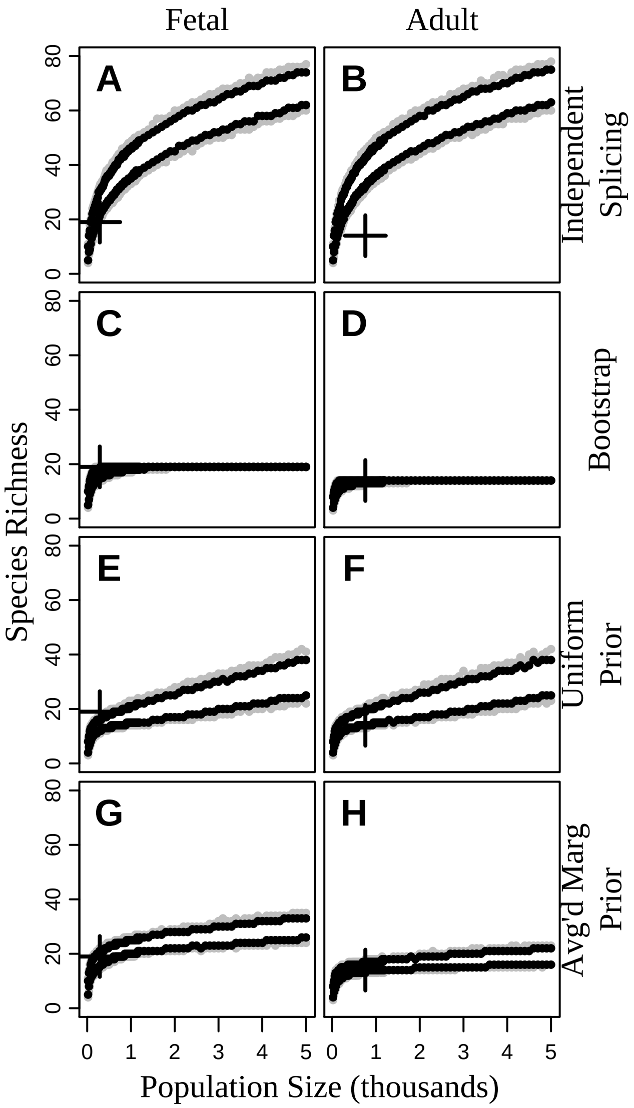

Figure S3: Species richness in observed and Monte Carlo-simulated cDNA populations. Monte Carlo populations of various sizes were sampled from four estimator distributions for each tissue. Estimators A-D were derived directly from the data: **A** and **B** were sampled from the expected distribution for independent splicing at the observed marginal frequencies of the individual loci (ϕ_v, equation 1 in the main paper); **C** and **D** were resampled from the observed frequencies of the splice variants. Estimators E-H were empirical-Bayes posteriors obtained with a uniform prior (**E**, **F**) or the "averaged-marginals" prior (**G**, **H**) discussed below. 1000 populations of each size were sampled. Black symbols mark the 5 and 95% quantiles, and gray mark the 1 and 99% quantiles. Crosses plot the observed values. This figure may be interpreted in two ways: (i) as a test of model adequacy; for example, independent splicing is insufficient to account for the observed adult population, and bootstrap models only the observed forms, and (ii) given an acceptable model it shows how many transcripts must be sampled to ensure that a desired number of forms represented. The bottom panels show that larger samples yield additional forms in both tissues, as generally expected, but progressively fewer in the adult than in the fetal.

Table S3: Marginal frequencies and entropies for splice configurations in table S1

C_j	**Δ25A**	**14**	**25C**	**26**	**30B**	**31A**	**34**	**35**	**38B**
$p_j(C_j)$									
Fetal	0.899	0.690	0.045	0.756	0.986	0.986	0.038	0	0.965
Adult	0.956	0.606	0.796	0.121	1.000	0.987	0.045	0.005	0.983
$H_j(C_j)$									
Fetal	0.327	0.619	0.184	0.556	0.074	0.074	0.162	0	0.152
Adult	0.180	0.671	0.506	0.369	0	0.069	0.184	0.031	0.086

The empirical Bayes estimate with uniform prior (E and F) captures the observed species richness only marginally in either population and gives an odd linear growth with sample size after an initial steep rising phase. The empirical Bayes estimate with averaged-marginals prior (G and H), brackets the observed values and shows a reasonable growth characteristic, with progressively diminishing yield, relatively flatter in the adult than the fetal, consistent with greater splicing linkage in the former. We sill discuss this estiamtor in greater detail. The mutual information analyses show that the overall observed splicing linkage is not high in either tissue, except for loci **25C** and **26** (Figure 1B in the main text). To a first approximation, the distribution of splice variants in both tissues follows the expected stochastic distribution for independent splicing of the separate loci (Figure S4). We may include this information in the prior distribution. Independent support for this comes also from Latour *et al.* (2004) and Monteil *et al.* (2000) who obtained a population of 68 long-range adult brain Ca_v3.1 cDNAs whose multinomial log-likelihood is consistent with our data, based on independent splicing at our observed marginal frequencies.

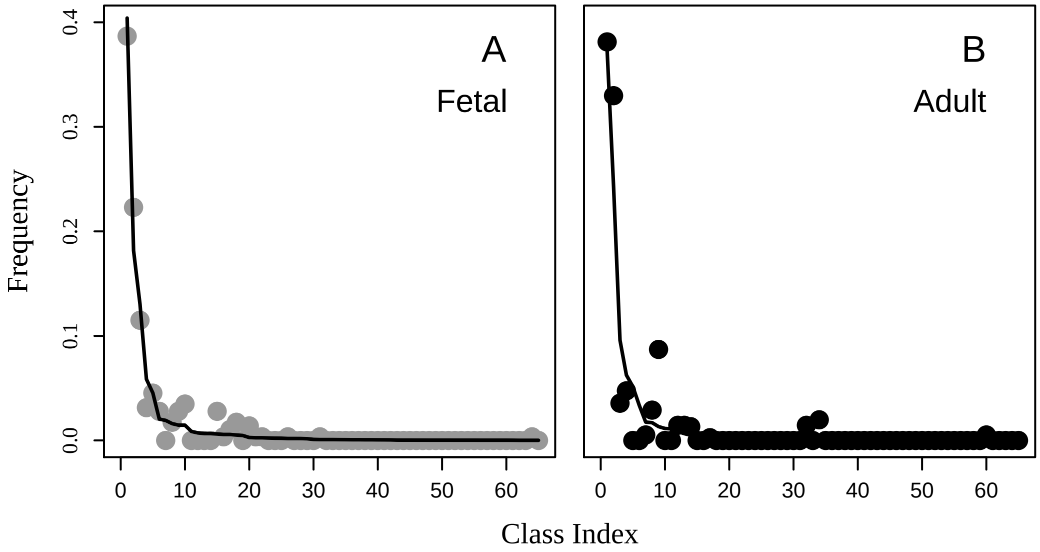

Figure S4: Splice variants ranked by expected frequency. The first 65 fetal splice variant classes are plotted in order of expected frequency given independent, stochastic splicing (**black curve**); a given class index does not generally refer to the same splice variant in the fetal and adult plots. Plot symbols indicate observed frequencies. All classes with more than one observed instance are plotted.

Several loci exhibit such a strong splicing bias (Figure S5 and table S3) that marginal frequencies cannot be estimated reliably with our sample size. Since these loci tend to yield the same configuration in both tissues, however, the tissue-averaged marginal frequencies were used for these loci (**30B**, **31A**, **34**, **35**, and **38B**), while tissue-specific values were used for the remaining loci (**14**, **25A**, **25B**, **26**). The stochastic expectation for independent splicing at these frequencies gives the tissue-specific 'independent-marginals' prior. As indicated in table S2, the fetal population conforms considerably to this prior, resulting in a high degree of shrinkage toward independence ($w = 0.34$). The adult frequencies are shrunk to a greater extent in this case ($w = 0.041$) than with the uniform prior, but markedly less than the fetal.

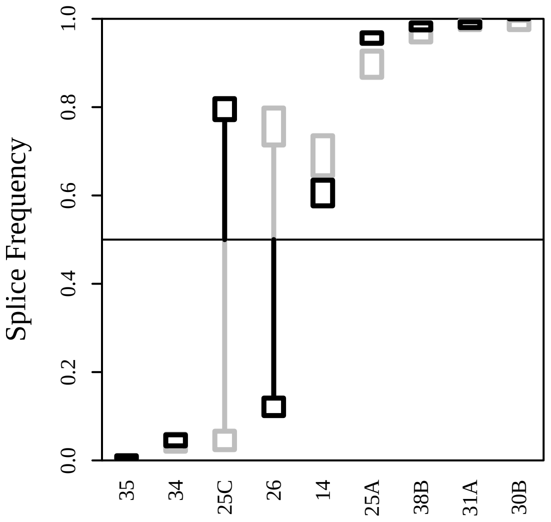

Figure S5: Marginal splicing probabilities. Boxes delimit the 95% confidence intervals for a binomial sample at the observed frequency in the fetal (gray) and adult (black) cDNA libraries. Vertical lines accentuate opposite-going developmental splicing shifts at loci **25C** and **26**.

We note that the high weight given to the fetal 'independent-marginals' prior, in contrast to the adult, reflects an important distinction between splicing of this gene in adult and fetal brain: fetal splicing linkage is relaxed compared to the adult, and this is of physiological importance. We emphasize that the resulting posterior distributions may very well reflect the true physiological parameters, but the fetal data are shrunk to such a degree that the observed frequencies no longer appear to be well represented in the posterior distribution (Figure S6A, blue symbols), and the pair-wise splicing linkage between loci **25C** and **26** is reduced in the posterior (Figure S2B). Unobserved variants are apparently overcompensated as well in both the fetal and adult populations (*c.f.* Figure S7, center column for each set of three priors).

To center our estimator better over the data, we should "back off" on the extent of shrinkage, and it is best to do so in a manner that is not preferential to one data set. If we simply average the observed fetal and adult marginal frequencies at each locus, we obtain a single 'tissue-averaged independent-marginals' prior that is roughly equidistant from both populations, resulting in a similar, small extent of shrinkage for both tissues (table S2). This yields an accurate representation of the observed classes (Figure S6A and C, green crosses) and a faithful reproduction of the observed pair-wise splicing correlations (Figure S2C). Thus the posterior distribution is largely a reflection of the properties of the observed data in both tissues and is therefore a conservative estimate, in that we do not depend heavily on the closeness of either population to its independent-splicing

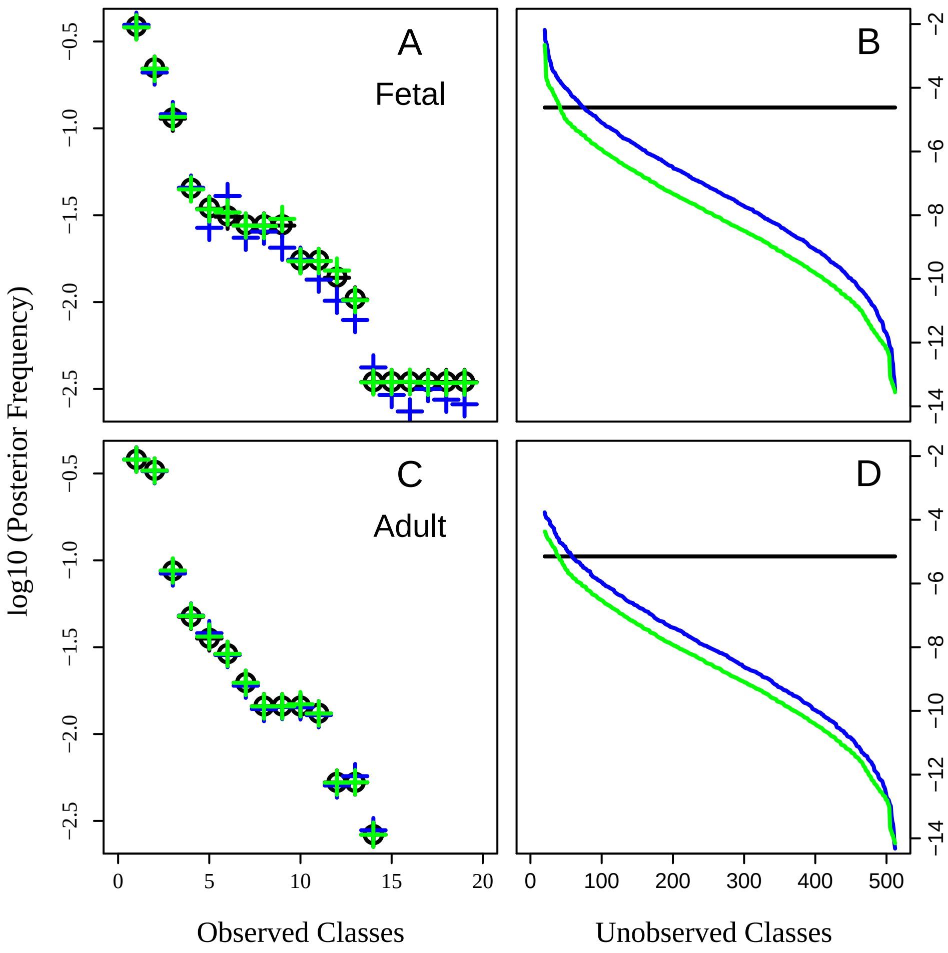

Figure S6: Empirical Bayes posterior distributions for three priors. Splice variant classes on the abscissa are listed in decreasing order of observed frequency (**A** and **C**) or expected frequency in the posterior distribution (**B** and **D**) for three priors: uniform (**black**), tissue-specific (**blue**), and averaged-marginals (**green**).

expectation. Unlike the uniform prior, however, both independent-marginals priors dampen contributions from highly unlikely splice configurations (compare either of the colored curves with the black curve in Figure S6B and D). The tissue-averaged prior improves on the tissue-specific prior by reducing the expected frequency of the first few unobserved classes (compare the two colored curves in Figure S6B and D).

This point is best illustrated in Figure S7. Each plot in this figure is a histogram of counts for a single splice variant in 1000 Monte Carlo populations sampled from the posterior estimate for one of three priors. The colored line demarks observed counts. The top row of plots in each set of three columns corresponds to the same observed splice variant (the one with the lowest counts in that tissue). The remaining plots in the column correspond to the first four unobserved classes, in order (downward) of decreasing posterior frequency. The tissue-specific prior over-estimates the first few unobserved classes, especially in the fetal samples. The averaged-marginals prior gives more expected behavior: the counts in the unobserved cells taper off more gradually in the fetal than the adult samples for this prior, consistent with less restrictive fetal splicing linkage. The uniform prior, by contrast, gives an unreasonably abrupt transition to very low counts in the unobserved

cells in both tissues, and all unobserved classes are in fact exchangeable.

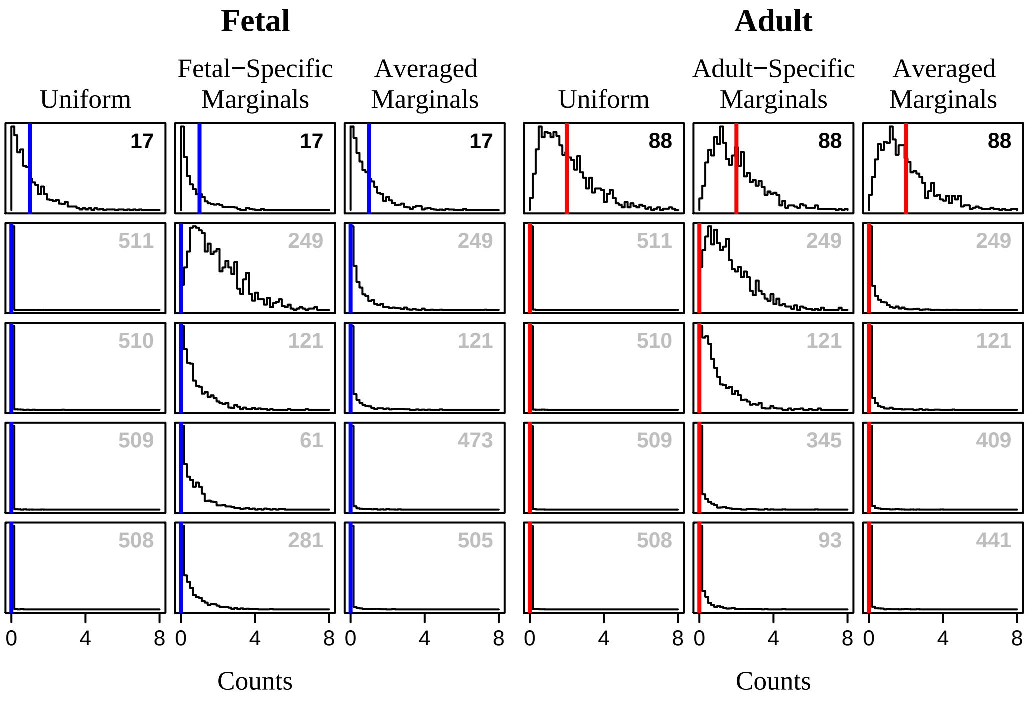

Figure S7: Low-frequency splice variants sampled from three estimators: details of the dependence on the prior. The top row depicts the least-frequent observed class. The remaining rows depict the four most frequent unobserved forms ranked in order (top to bottom) of decreasing frequency in the posterior. Fractional counts arise because the posterior distribution is continuous (Dirichlet).

It might be argued that by averaging out the marginal frequencies of **25C** and **26** we are neglecting the actual observation that these segments change inversely during development. This developmental switch has been noted not only by us, but by others as well (Monteil *et al.*, 2000; Latour *et al.*, 2004). We may account for this in the averaged-marginals prior in various ways. One possiblitiy is to assign a higher marginal frequency (0.75) to the 'major' configurations (**-25C and +26** in fetal and **+25C and -26**) and the complement one (0.25) to the minor configurations. This yields results that do not differ substantially from the tissue-specific independent-marginals prior. Alternatively, we may use the average observed 'major' and 'minor' frequencies in the same way. This produces results midway between that independent-marginals and averaged-marginals priors.

3.2 Assessing goodness of fit

An important criterion for selecting a data-based prior is that the experimental population should occur near the center of the posterior distribution. The distance of a sampled population $Y = y_1, y_2, \ldots, y_k$ from the experimental 'target' $X = x_1, x_2, \ldots, x_k$ is measured by the integrated squared error,

$$ISE = \sum_i (x_i - y_i)^2 \qquad \text{(S2)}$$

Figure S8 plots the ISE for each of 1000 populations sampled from estimators derived from three priors. These priors are mostly indistinguishable by this criterion. A related measure is chi-square, in which each term of the sum (S2) is divided by the variance of y_i. This measure is inapplicable

for the ‘independent-marginals’ priors, because the denominator tends to zero for the extremely unlikely classes (Figure S9).

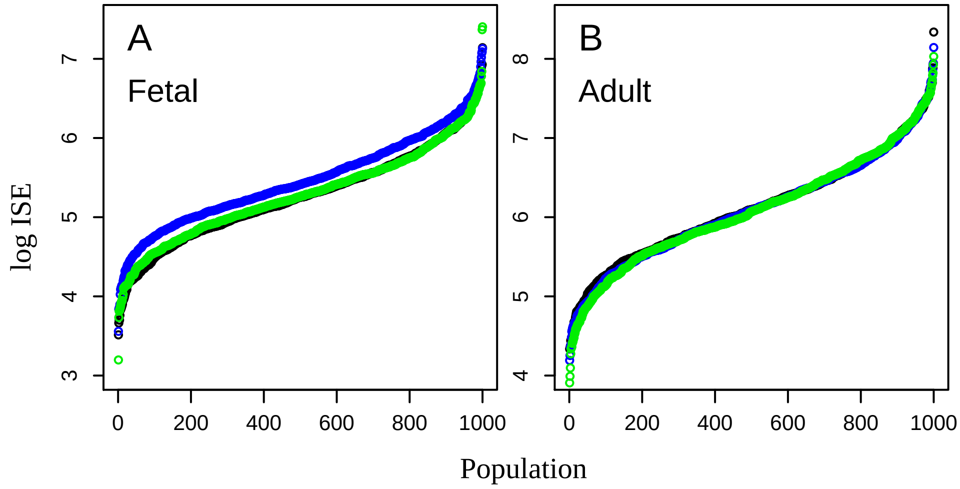

Figure S8: Overall goodness of fit measured by integrated squared error. Log(ISE) is plotted for each of 1000 populations sampled by Monte Carlo from the posterior distribution for each of three priors: uniform (**black**), tissue-specific (**blue**), and averaged-marginals (**green**).

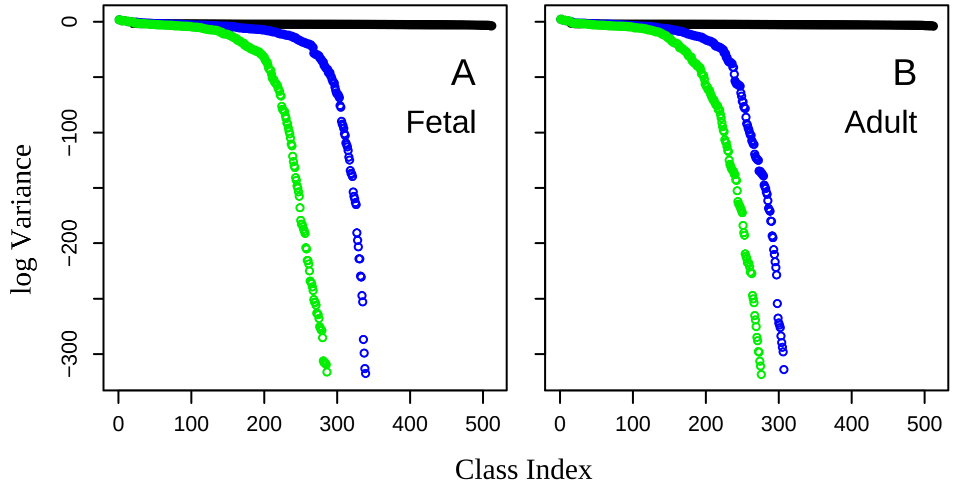

Figure S9: Dependence of the variance of different splice forms on the prior distribution. Log(Variance) is plotted in order of decreasing frequency in the posterior, for each of three priors: uniform (**black**), tissue-specific (**blue**), and averaged-marginals (**green**). Note that the variance of the uniform prior is not everywhere constant, but slightly elevated among the first few (observed) classes.

We are at present interested primarily in the statistical significance of differences in splicing-linkage structures between two populations, and not in making the most precise measurement of the properties of any particular population. Nonetheless, the bias-variance trade-off achieved by usual mean squared error measures of fit did not give satisfactory results in distinguishing priors that are clearly differentiable by direct inspection of clockplots, for example (Figure S2). The ‘accuracy’ index ($\mathcal{A}$), summed over all species in a population, is a very simple metric that works well for this purpose. Figure S10 plots the mean accuracies of all splice variants (squares) or just the observed

subset (circles) for populations sampled from 14 different posterior distributions, derived from six different priors by various degrees of shrinkage. The 'independent-marginals' priors are the most accurate, overall, while the uniform prior is satisfactory for the observed variants only. Posteriors 7-10 derive from four 'independent-marginals' prior variations by direct application of equations (S1). The accuracy drops off with posteriors 8-10, especially for the observed fetal variants. This is because a closer fit of the prior to the likelihood results in larger values of K in equation (S1c) (the 'Bishop' weight) and thus greater shrinkage toward independence, away from the largest observed values. The first six posteriors (except for #5) derive from independent-marginals priors, but with the prior weight reduced 'manually' to varying extents below the Bishop value. The observed accuracies plateau near the level set by the Bishop-weighted averaged-marginals prior (#7). The accuracies for the unobserved variants increase slightly because higher weight is given to the observed frequency of zero, resulting in a more compact distribution for these classes in the Monte Carlo populations. We emphasize that the apparent improvement in accuracy that results from decreasing the prior weight below the Bishop weight is mainly due to reducing the probabilities of unobserved variants. This is not justified in general, as equations (S1) optimize the weight for the choice of prior (Bishop, Fienberg et al. 1975). Furthermore, decreasing the prior weight in this way does not have a significant effect on the relative magnitudes of the log-linear coefficients for the interaction terms (Figure S12, discussed below).

The 'averaged-marginals' prior is a very simple, intuitive prior that, with straightforward Bishop shrinkage, (1) reproduces the observed pair-wise splicing correlations in the posterior distribution, (2) is well centered with respect to both the complete set of variants as well as just the observed subset, and (3) does not incorporate the plainly unreasonable assumption that all unobserved variants are equally likely, and gives a justifiable distribution of prior probabilities for those variants.

Figure S11 presents fetal/adult comparative spliceprints from Monte Carlo populations sampled from posteriors derived by Bishop shrinkage from several of the priors just discussed. The shape of the profiles is fairly stable across priors, except in two cases (panels E and F, the tissue-specific marginals prior and a close variant) where increased shrinkage toward independence produces a flattened fetal profile (compare Figure S2B).

Figure S12 is an assessment of the effects of shrinkage on the relative magnitudes of coefficents. It is a version of Figure 8 in the main text, in which mean coefficient magnitudes are *relative* to order-1 (cardinality-2) interactions. Grayscale histograms correspond to Bishop shrinkage according to equations (S1) (yielding $w = 0.022$ in fetal and 0.0003 in adult), while blue shading corresponds to linear shrinkage at a constant, lower extent ($w = 0.002$ in both fetal and adult). Relative magnitudes are stable to variation in the extent of shrinkage with the same prior, though reducing the shrinkage gives smaller variances.

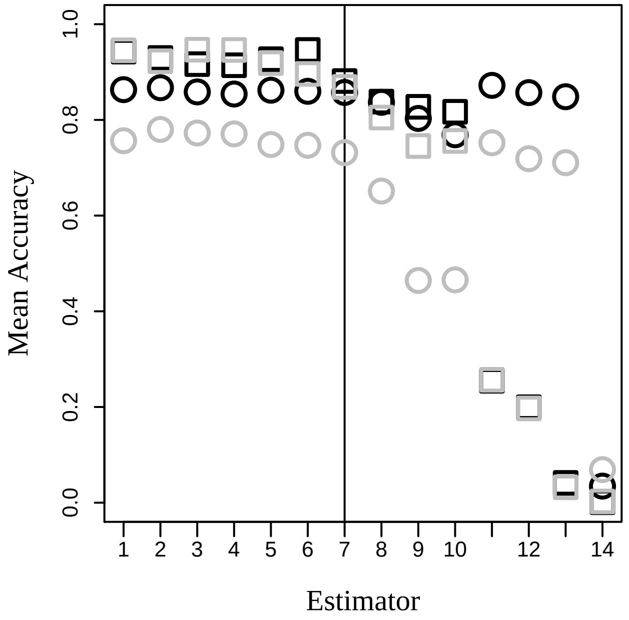

Figure S10: Estimator assessment. For each of 14 estimators, the mean accuracy ($\mathcal{A}$, *c.f. methods*) is plotted for all 512 classes (squares) or for just the observed subset (circles), using 1000 populations sampled by Monte Carlo from the posterior distribution, **p***, derived by equation (S1a). Gray = fetal, Black = Adult. The three priors discussed most extensively are uniform (13), tissue-specific marginals (10), and averaged-marginals (7), all with weights determined by equation (S1c). Estimators 1 through 6 are obtained by 'manually' reducing the extent of shrinkage, over-weighting the data relative to to the Bishop weight, w in equation (S1c). This converges to the bootstrap at low weight, has little effect with a sound prior, and defeats the Bishop weighting rationale. The others are obtained as follows: Where indicated, sensitivity to shrinkage is tested by specifying K or w directly, rather than applying equation (S1c). Prior **1**: Tissue-specific prior with $w = 1/N$; **2-9**: Averaged-marginals prior with (2) $w = 1/758$, (3) $w = 1/500$, (4) $w = 1/287$, (5) a single "minor" frequency—equal to the average of the observed frequencies of **25C** in the fetal population and **26** in the adult— assigned to those two configurations, the corresonding average "major" frequency assigned to fetal **26** and adult **25C**, and K set to $1/25$ of the value determined by applying equation (S1c) (compare prior 9), (7) (Averaged marginals for all loci), (8) the minor and major frequencies (defined in prior 6) set to 0.75 and 0.25, respectively, (9) Same as prior 6, but with K determined by equation (S1c); **10**: tissue-specific marginals; **11**: Uniform, $K = 1$; **12**: Random—512 draws from a uniform distribution on $[0,1]$; **13**: Uniform; **14**: Unique random prior for each Monte Carlo population. Priors 6 and 8 incorporate the **25C/26** developmental switch into the averaged-marginals prior, progressively de-emphasizing the specific observed frequencies at those loci.

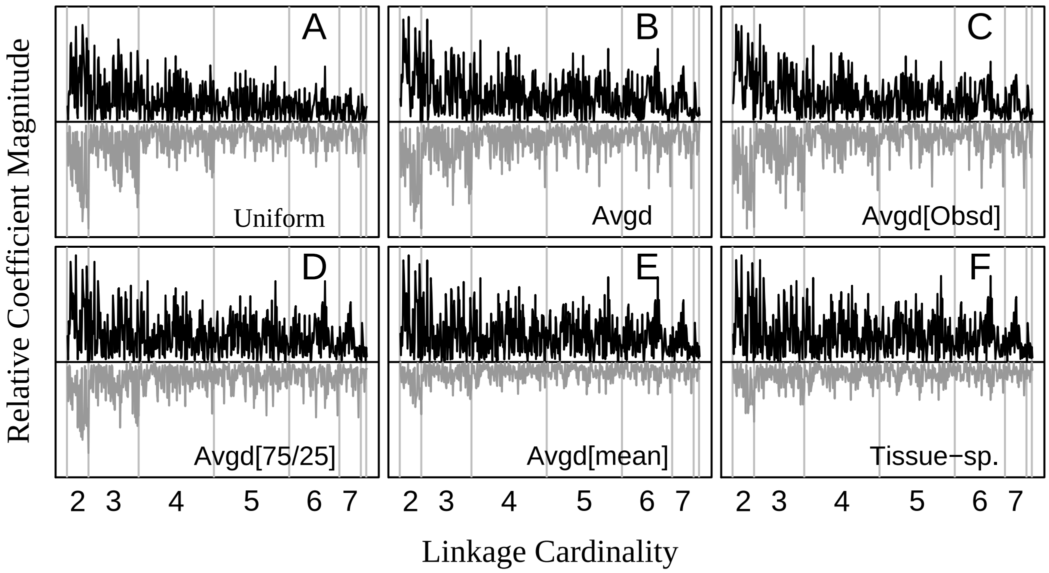

Figure S11: Comparative spliceprints for various fetal and adult estimators. Adult values (black) are plotted above and fetal values (gray) below the midline in each plot. Each trace plots the mean of the coefficients derived from loglinear model fits to Empirical Bayes estimates from 1000 MC populations. Priors are numbers 13 (**A**), 7 (**B**), 5 (**C**), 8 (**D**), 6 (**E**), and 10 (**F**) of Figure S10. Plots B-E use variants of the averaged-marginals prior. Notes in brackets indicate how minor/major frequencies at **25C** and **26** were obtained (see figure S10 legend for priors 5, 6, and 8).

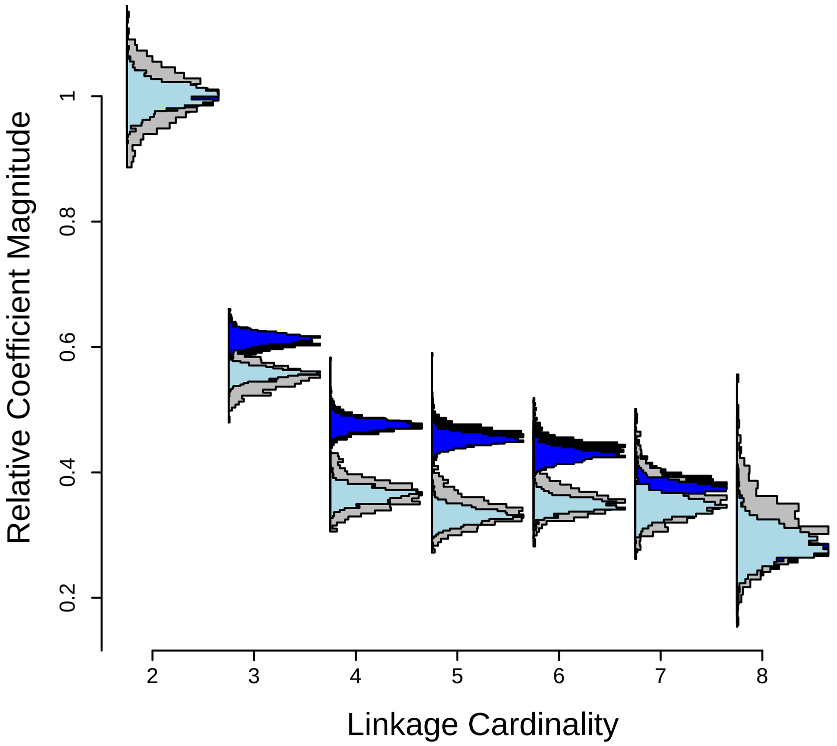

Figure S12: Distribution of cardinality-averaged loglinear coefficients. Relative coefficient magnitudes are stable to differing extents of shrinkage.

References

Bishop, Y.M.M., Fienberg, S.E. and Holland, P.W. (1975) Discrete multivariate analysis: Theory and practice. Cambridge: MIT Press.

Carlin BP, Louis TA. (1996) Bayes and empirical Bayes methods for data analysis. 1st ed. London, New York: Chapman and Hall

Emerick, M.C., Stein, R., Kunze, R., McNulty, M., Regan, M.R., Hanck, D.L., and Agnew, W.S. (2005) Profiling the Array of Ca_v3.1 Variants from the Human T-type Calcium Channel Gene CACNA1G: alternative structures, developmental expression and biophysical variations. *Proteins: struct. funct. bioinform.*, bioinform., Published Online: 2 May 2006..

Monteil A, Chemin J, Bourinet E, Mennessier G, Lory P, Nargeot J (2000) Molecular and functional properties of the human alpha(1G) subunit that forms T-type calcium channels. J Biol Chem 275: 6090–6100.

Latour I, Louw DF, Beedle AM, Hamid J, Sutherland GR, and G. W. Zamponi GW (2004) Expression of T-type calcium channel splice variants in human glioma. Glia 48: 2, pp. 112–119.
